# Supplementary material for: Moving forward through consensus: a national Delphi approach to determine the top research priorities in prostate cancer in Uganda
Source: BMJ Open. 2023 Nov 30;13(11):e075739. doi: 10.1136/bmjopen-2023-075739 (PMC10689405; doi:10.1136/bmjopen-2023-075739)
Supplement: Supplementary data [file bmjopen-2023-075739supp001.pdf]

Guidance Document for Completion of the APC-DOUG Survey

The guidance below relates to Round 1 of the Delphi process.

The aim of Round 1 is to prioritize for further research and intervention development, the major barriers that exist in accessing prostate cancer care. We have considered three aspects to the delays that may occur in receiving a diagnosis and/or treatment.

Delay 1 (seeking care): This is the delay in recognizing illness and deciding to seek appropriate medical help outside the home.

Delay 2 (reaching care): This is the delay in reaching an appropriate health facility.

Delay 3 (receiving quality care): This is the delay in receiving quality care after reaching the health facility.

We are kindly asking you to review and answer the questions below based on your experience.

Instructions:

1. There are 21 statements identified from a review of the wider empirical literature as a reason for delay in accessing cancer care.
2. We want to understand how important or relevant these reasons for delays are with respect to prostate cancer in Uganda
3. Each statement will be scored out of 20 based on 4 assessment domains a. feasibility to study this issue; b. scale of the issue; c. impact of the barrier on prostate cancer outcomes; d. feasibility of addressing this barrier
4. Please score each domain on each delay out of 5, with 5 being the highest (most important) and 1 being the least (important).
5. The statements assigned the highest scores will be considered as main priorities for round 2 of the Delphi process.

If you have any questions, please contact Dr Amos D. Mwaka email: mwakaad@yahoo.com or Andrew S. Ssemata email: andrew.ssemata@mrcuganda.org.

**Reason for delay**	**Feasible (Easy to measure or investigate this reason for delay)**	**Large scale (This delay affects a significant proportion of prostate cancer patients)**	**High Impact (This delay is a significant or avoidable cause of death or disability from prostate cancer)**	**Modifiable (The reason identified can be readily changed to improve care)**
	Please score each theme for each statement with a score of 1 – 5 based on the explanation in brackets (1 is lowest and 5 is highest)			
1. Accessibility of care (long distance/travel times to access specialist services)				
2. Lack of awareness of cancer as a disease and recognition of symptoms				
3. Poor healthcare literacy (when, how and where to seek services)				
4. Preference for traditional, complementary, and alternative medicines				
5. Lack of trust in healthcare system and patients' citizens' rights (perceived quality; attitudes of healthcare workers; previous bad experience e.g., patients being turned away or refusal to refer; adequate consent)				
6. Personal and professional obligations (financial and social implications to the patient and their families of seeking care and undergoing treatment)				
7. Stigma associated with a cancer diagnosis or severe illness/ Fears and beliefs around cancer				
8. Lack of social capital (relationships, support from family, friends, colleagues)				

**Reason for delay**	**Feasible (Easy to measure or investigate this reason for delay)**	**Large scale (This delay affects a significant proportion of prostate cancer patients)**	**High Impact (This delay is a significant or avoidable cause of death or disability from prostate cancer)**	**Modifiable (The reason identified can be readily changed to improve care)**
9. Misdiagnosis of cancer (at lower system levels e.g. primary care, district hospital)				
10. Difficulties with healthcare coordination between regions and hospitals as patients referred for specialist investigation and treatment				
11. Lack of diagnostic services (X-ray, Ultrasound, labs (e.g. PSA testing, biopsy facilities)				
12. Lack of availability of critical medicines				
13. Lack of radiotherapy options (brachytherapy/ teletherapy)				
14. Lack of critical surgical supplies				
15. Cost of accessing healthcare (e.g., cost of accommodation and transport needed to receive treatment from centralized services)				
16. Cost of diagnostic investigations				
17. Cost of treatments e.g. surgery, radiotherapy. Hormone therapy				
18. Lack of workforce (basic numbers low and inadequately trained staff)				
19. Patient fitness and treatment toxicity				
20. Communication/language barriers between health care staff and patients				
21. Staff motivation and burnout				

Ssemata AS, *et al. BMJ Open* 2023; 13:e075739. doi: 10.1136/bmjopen-2023-075739

Ssemata AS, *et al. BMJ Open* 2023; 13:e075739. doi: 10.1136/bmjopen-2023-075739
