# Supplementary material for: Moving forward through consensus: a national Delphi approach to determine the top research priorities in prostate cancer in Uganda
Source: BMJ Open. 2023 Nov 30;13(11):e075739. doi: 10.1136/bmjopen-2023-075739 (PMC10689405; doi:10.1136/bmjopen-2023-075739)
Supplement: Author's manuscript [file bmjopen-2023-075739.draft_revisions.pdf]

BMJ Open is committed to open peer review. As part of this commitment we make the peer review history of every article we publish publicly available.

When an article is published we post the peer reviewers' comments and the authors' responses online. We also post the versions of the paper that were used during peer review. These are the versions that the peer review comments apply to.

The versions of the paper that follow are the versions that were submitted during the peer review process. They are not the versions of record or the final published versions. They should not be cited or distributed as the published version of this manuscript.

BMJ Open is an open access journal and the full, final, typeset and author-corrected version of record of the manuscript is available on our site with no access controls, subscription charges or pay-per-view fees (http://bmjopen.bmj.com).

If you have any questions on BMJ Open's open peer review process please email info.bmjopen@bmj.com

BMJ Open

Moving forward through consensus: a national Delphi approach to determine the top research priorities in prostate cancer in Uganda.

Journal:	*BMJ Open*
Manuscript ID	bmjopen-2023-075739
Article Type:	Original research
Date Submitted by the Author:	17-May-2023
Complete List of Authors:	Ssemata, Andrew; MRC/UVRI and LSHTM Uganda Research Unit, Social Aspects of Health Across the Lifecourse; London School of Hygiene & Tropical Medicine, Department of Global Health and Development<br>Muhumuza, Richard; MRC/UVRI & LSHTM Uganda Research Unit, Social Aspects of Health across the Lifecourse<br>Seeley, Janet; MRC/UVRI and LSHTM Uganda Research Unit, Social Aspects of Health across the Lifecourse; London School of Hygiene and Tropical Medicine, Department of Global Health and Development<br>Lombe, Dorothy; MidCentral District Health Board, Radiation Oncology<br>Mwamba, Monde; University of Zambia<br>Msadabwe, Susan; Cancer Diseases Hospital<br>Mwaka, Amos; Mulago Hospital/Makerere University, Department of Internal Medicine; Gulu University, Department of Medicine, Faculty of Medicine<br>Aggarwal, Ajay; London School of Hygiene and Tropical Medicine Faculty of Public Health and Policy, Health Services Research & Policy; King's College London
Keywords:	Prostate, Cancer pain < ONCOLOGY, Primary Health Care, Prostate disease < UROLOGY, Quality in health care < HEALTH SERVICES ADMINISTRATION & MANAGEMENT

SCHOLARONE™
Manuscripts

For peer review only - http://bmjopen.bmj.com/site/about/guidelines.xhtml

I, the Submitting Author has the right to grant and does grant on behalf of all authors of the Work (as defined in the below author licence), an exclusive licence and/or a non-exclusive licence for contributions from authors who are: i) UK Crown employees; ii) where BMJ has agreed a CC-BY licence shall apply, and/or iii) in accordance with the terms applicable for US Federal Government officers or employees acting as part of their official duties; on a worldwide, perpetual, irrevocable, royalty-free basis to BMJ Publishing Group Ltd ("BMJ") its licensees and where the relevant Journal is co-owned by BMJ to the co-owners of the Journal, to publish the Work in this journal and any other BMJ products and to exploit all rights, as set out in our licence.

The Submitting Author accepts and understands that any supply made under these terms is made by BMJ to the Submitting Author unless you are acting as an employee on behalf of your employer or a postgraduate student of an affiliated institution which is paying any applicable article publishing charge ("APC") for Open Access articles. Where the Submitting Author wishes to make the Work available on an Open Access basis (and intends to pay the relevant APC), the terms of reuse of such Open Access shall be governed by a Creative Commons licence – details of these licences and which Creative Commons licence will apply to this Work are set out in our licence referred to above.

Other than as permitted in any relevant BMJ Author's Self Archiving Policies, I confirm this Work has not been accepted for publication elsewhere, is not being considered for publication elsewhere and does not duplicate material already published. I confirm all authors consent to publication of this Work and authorise the granting of this licence.

For peer review only

Word count: 2955

Moving forward through consensus: a national Delphi approach to determine the top research priorities in prostate cancer in Uganda.

Authors:

Andrew Sentoogo Ssemata[1, 2*], Richard Muhumuza[1], Janet Seeley[1, 2], Dorothy Chilambe Lombe[3], Monde Mwamba[4], Susan Msadabwe[5], Amos Deogratius Mwaka[6,7], Ajay Aggarwal[8,9]

1. **Affiliations:** The Medical Research Council/ Uganda Virus Research Institute and London School of Hygiene & Tropical Medicine (MRC/UVRI & LSHTM) Uganda Research Unit, Entebbe, Uganda.
2. Department of Global Health & Development, London School of Hygiene and Tropical Medicine, London, UK.
3. Radiation Oncology, Te Whatu Ora Health NZ MidCentral, Palmerston North, New Zealand.
4. ZAMBART, Lusaka, Zambia.
5. Department of Radiation Oncology, Cancer Diseases Hospital, Lusaka, Zambia.
6. Department of Medicine, College of Health Sciences, Makerere University, Kampala, Uganda.
7. Department of Medicine, Faculty of Medicine, Gulu University, Gulu, Uganda
8. Department of Health Services Research and Policy, London School of Hygiene and Tropical Medicine, London, UK.
9. King's College London, London, UK.

***Correspondence to:** andrew.ssemata@mrcuganda.org

For peer review only

Abstract

Objective: To describe the health system, socioeconomic and clinical factors that influence access to and outcomes of care for prostate cancer (PC) in Uganda.

Design: Qualitative Delphi technique

Setting: Government and private-not-for-profit hospitals

Methods: We applied a two-stage modified Delphi technique to identify the consensus view across cancer experts. In the first round, experts received a questionnaire containing 22 statements drawn from a systematic review of the literature focusing on identifying the reason for the delay in accessing cancer care. Round 2 comprised 18 statements considered the greatest priority from round 1.

Results: We found that the top five research priority areas arise from challenges including: (i) lack of Diagnostic services - Ultrasound, laboratory tests, and biopsy facilities; (ii) High costs of services e.g., surgery, radiotherapy, hormone therapy are unaffordable to most patients, (iii) Lack of critical surgical supplies and essential/critical medicines, (iv) Lack of awareness of cancer as a disease and low recognition of symptoms, (v) Low Healthcare literacy. The lack of critical surgical supplies, high costs of diagnostic investigations and treatments were ranked highest in order of importance in round one. Round two of the survey also revealed lack of diagnostic services, unavailability of critical medicines, lack of radiotherapy options, high costs of treatments and lack of critical surgical supplies as the top priorities.

Conclusion: These research priority areas ought to be addressed in future research to improve prompt prostate cancer diagnosis and care in Uganda. There is need to improve the supply of high-quality affordable anticancer medicines provided timely to prostate cancer patients so as to improve the survivorship from the cancer.

Keywords: Prostate cancer, delays, health care, Uganda, Delphi technique

Article Summary

Strengths and limitations of this study

- This is a novel Delphi study exploring research priorities for prostate cancer research in Uganda from various stakeholders in cancer care.

For peer review only - http://bmjopen.bmj.com/site/about/guidelines.xhtml

- The study was informed by a recent systematic review which provided insight into the statements explored in the Delphi technique.
- Participants were recruited from various clinical, government aided and private settings and geographical locations giving representativeness of the participants.
- A limitation to this study was the low response rates in round 1 and round 2 as most stakeholders were not familiar with the Delphi technique, or how it works.

For peer review only

Introduction

In 2020, there were 19.3 million new cancer cases and 10 million cancer deaths worldwide.[1] Prostate cancer is among the top 3 most common incident cancers in men (1.3 million incident cases) and the leading cause of cancer-related deaths for men in 56 countries including Uganda.[2]

A recent review has presented a worrying disparity in the mortality to incidence ratio from prostate cancer at 90% in Africa compared to 10% in North America.[3] For a curable cancer these figures are stark. In the low- and middle-income countries (LMICs), one of the challenges facing cancer control is the late diagnosis with advanced stage cancers at diagnosis.[4-6] The reasons that contribute to delayed diagnoses and advanced stage at presentation include delayed recognition of symptoms and signs of cancers by patients and primary healthcare professionals, inadequate health and cancer information for patients, poor continuity of care, lack of facilities for biopsy and histological diagnoses, costs and availability for treatments including drug therapies.[6-8]

One of the key challenges to delivering timely prostate cancer care is that the condition is largely asymptomatic in its early stages. African men also face much higher prostate cancer frequency, severity, earlier age of disease and greater risk of death than is seen in Caucasian populations, leading to much greater economic impacts on societies.[9]

For peer review only - http://bmjopen.bmj.com/site/about/guidelines.xhtml

In Uganda, there has been much emphasis on cancers affecting women, especially breast and cervical cancers.[10-12] Little attention has been given to the cancers affecting men especially prostate cancer that has been increasing in incidence over the recent decades.[13-15] This apparent neglect and lack of data for planning for cancers affecting men has generated the need to prioritise key areas for research.

We present findings from the African Prostate Cancer - Disparities in Outcomes in Uganda (APC-DOUG) study whose aim was to describe health system, socioeconomic and clinical factors that influence access to and outcomes of care for prostate cancer across hospitals in Uganda. The purpose of this Delphi process was to prioritise key areas for research in prostate cancer in the Ugandan context by establishing the major barriers to seeking cancer care, reaching cancer care, and receiving high quality cancer care. By defining future research priorities and investigations, this will help to support the future development of health system interventions to reduce inequalities and improve prostate cancer outcomes in hospitals across Uganda.

Methods

The Delphi technique is a relevant source of evidence in health care research foundational in the formation of consensus or the exploration of a field beyond existing knowledge.[16] It has been used in medicine to develop research priorities.[16-18]

A structured 2-round modified Delphi technique with Ugandan clinical and health system research experts involved in prostate cancer care was conducted between December 2021 and July 2022. The Delphi technique was used to elicit consensus on the key research priorities in relation to the delays that may occur in receiving a diagnosis and/or treatment based on the Three Delays framework.[19 20] The "Three Delays" Framework has been effective as a rapid health system assessment tool to understand complex multi-factorial problems affecting access to care.[20] The Three delays framework focused on delays to: (i) Seeking care - delays in recognizing illness and deciding to seek appropriate medical help outside the home; (ii) Reaching care - delays in reaching an appropriate health facility and (iii) Receiving quality care - delays in receiving quality care after reaching the health facility.[21]

For peer review only - http://bmjopen.bmj.com/site/about/guidelines.xhtml

Development of the survey

The Delphi survey was informed by two systematic reviews undertaken by the study team appraising primary quantitative,[15] and qualitative (Mwamba et al., under peer review) research studies that had sought to identify the factors influencing delays in cancer diagnosis and treatment as well as receiving quality care in Sub-Saharan Africa. From this literature review 22 key factors contributing to delays were identified and represented priority areas for further investigation. These were translated into written statements that formed part of the Delphi consensus exercise for prioritizing research in prostate cancer in Uganda (supplemental appendix 1).

Data collection

Data collection for round 1 of the Delphi process was conducted between December 2021 and February 2022 and round 2 of the Delphi process between March and July 2022. Participants were selected to ensure representation from different regions, health care sectors (primary and secondary); specialist disciplines (urology, oncology); academia and public health as well as patient representatives. Lists of participants to approach from both public and private health facilities in Kampala, Wakiso, and Mukono districts were developed by the planning committee which comprised two oncologists, and two health services research methodological experts who oversaw the design, execution, and analysis of all phases of the study.

At present there is only one major comprehensive cancer treatment centre in Uganda (the Uganda Cancer Institute, based at Mulago Hospital) with two active regional bases (in Mbarara, western Uganda, and Gulu; Northern Uganda),[6] and representation from each of these were sought. A snowballing approach was also undertaken where participants recommended additional stakeholders for participation. Agreement was reached regarding participant selection, consensus thresholds, and survey format and question structure. For round 1, we approached participants from the Uganda Cancer Institute, government hospitals, Private-for-profit hospitals, Private-not-for-profit hospitals, Ministry of Health, and a university.

Procedure

For peer review only - http://bmjopen.bmj.com/site/about/guidelines.xhtml

Experienced social science and clinical researchers conducted the data collection using a survey questionnaire. Participants were approached by a formal invitation to participate by email correspondence followed by a phone call and for some participants a face-to-face meeting. For those willing to participate, we collected demographic data, and a structured questionnaire was hand delivered to the participants. This was followed up with a reminder phone call within a four-week period to ensure completion of the question. Two sequential rounds of questionnaires were used for this Delphi process (Supplemental appendix 1 and 2).

Round 1 consisted of scoring the 22 statements using a 4-point scale. Each statement was assigned a score (out of 20) based on the four criteria, each scored out of 5, as below:

- **Feasible** – how easy is it to measure or assess this reason for the delay?
- **Large scale** – does this factor affect a considerable proportion of men with prostate cancer.
- **High Impact** –is this factor a significant cause of death or disability from prostate cancer?
- **Modifiable** – can this factor be reasonably addressed to improve the care of men with prostate cancer.

Once all the questionnaires for round 1 were received, we compiled the rankings for each statement. Based on the criteria by Schneider et al,[22] statements were selected as follows. Statements where 70% of participants gave a score of ≥15 were included as research priorities. Statements for which <30% of participants gave a score of ≥15 were excluded at this stage.[22] The rest of the statements went forward to the second round of the Delphi consensus process. The information provided in the round 1 of the Delphi technique was collated and summarized in REDCap software,[23] to enable formal analysis, and to formulate a second questionnaire with fewer statements based on the selection criteria above.

Round 2 of the Delphi included 18 statements. An electronic questionnaire designed in REDCap was generated and emailed to 42 participants. Participants were followed up with a reminder phone call within a four-week period and through face-to-face meetings by the study team. Recurrent emails and phone calls were made to those who had been contacted and had not completed the survey.

Ethical considerations

For peer review only - http://bmjopen.bmj.com/site/about/guidelines.xhtml

This study was performed in line with the principles of the Declaration of Helsinki. Ethical approval to conduct the Delphi technique was obtained from the Uganda Virus Research Institute Research Ethics Committee (UVRI-REC) Ref: GC/127/21/09/859 and the Uganda National Council for Science and Technology (UNCST) Ref: HS1790ES and the London School of Hygiene and Tropical Medicine Ref: 26672. Each participant provided written informed consent before any study procedures were conducted and were reimbursed for their time and participation in the study. Each participant was provided a time compensation of 30,000 Uganda shillings at the end of each completed questionnaire.

Results

Ten and twelve respondents completed the survey questionnaires in round 1 and 2 respectively. In round 1, a total of 30 clinicians were invited through email and face to face meetings, of which 10 (34%) participated and completed the survey. The majority of the participants in this round were oncologists $n = 4$ (40%) and cancer researchers $n = 3$ (30%). Most of the participants worked in a cancer care facility. In round 2, a total of 42 clinicians were invited through email, online and face to face meetings, of which 12 (29%) participated and completed the survey. Most of the participants in this round were radiologists $n = 3$ (25%) and general practitioners $n = 3$ (25%). Most of the participants in round 2 operated from a general health care setting. Of the 22 people who participated in round 1 and 2, three participated in both rounds and seven participants participated in round 1 only. The characteristics of the respondents in Rounds 1 and 2 are presented in table 1.

Table 1 showing characteristics of participants in round 1 and 2 of the Delphi process.

	**ROUND 1**		
	**Gender**	**Profession**	**Organisation**
1	Male	Oncologist	Cancer Institute, Kampala
2	Male	Oncologist	Cancer Institute, Kampala
3	Male	Oncologist	Cancer Institute, Kampala
4	Male	Cancer researcher and administrator	Cancer Institute, Kampala
5	Male	Oncologist	Cancer Institute, Kampala
6	Male	Public health specialist and policy expert	University/ Ministry of Health, Kampala

For peer review only - http://bmjopen.bmj.com/site/about/guidelines.xhtml

7	Male	Medical doctor/ General practitioner	Government hospital, Kampala
8	Female	Researcher and Clinical officer	Government hospital, Kampala
9	Male	Urologist	Cancer Institute, Kampala
10	Female	Surgeon and Cancer researcher	Government hospital, Kampala
	**ROUND 2**		
1	Female	Urologist	Private for-profit hospital, Kampala
2	Female	Surgeon and Cancer researcher	Government hospital, Kampala
3	Female	Researcher and clinical officer	Government hospital, Kampala
4	Male	Medical doctor/ General practitioner	Government hospital, Kampala
5	Male	Urologist	Government hospital, Kampala
6	Male	Medical doctor/ General practitioner	Private not-for-profit hospital, Kampala
7	Female	Radiologist	Private not-for-profit hospital, Kampala
8	Female	Radiologist	Private not-for-profit hospital, Kampala
9	Male	Surgeon	Government hospital, Mukono
10	Male	Medical doctor/ General practitioner	Government hospital, Mukono
11	Male	Surgeon	Private for-profit hospital, Kampala
12	Male	Radiologist	Private not-for-profit hospital, Kampala

Following the first round of the Delphi technique, we excluded four statements of research priorities from the second-round questionnaire where <30% of participants had given them a score less than 15. These statements were related to barriers such as staff motivation, inadequate training etc. Two of the barriers scored greater than 70% and were to be included within our final selection of research priorities. These were "*lack of awareness of cancer as a disease*" and "*poor health care literacy*" (table 2).

Table 2: Ranking of Round 1 questionnaire statements.

For peer review only - http://bmjopen.bmj.com/site/about/guidelines.xhtml

	Statement	Ranking (% of participants scoring reason >=15)
1	Lack of awareness of cancer as a disease and recognition of symptoms	90
2	Poor Healthcare literacy (when, how and where to seek services)	90
3	Lack of critical surgical supplies	70
4	Cost of diagnostic investigations	70
5	Cost of treatments e.g., surgery, radiotherapy, hormone therapy	70
6	Lack of Workforce (basic numbers low and inadequate training of staff)	70
7	Accessibility of care (long distance/travel times to access specialist services)	60
8	Difficulties with healthcare coordination between regions and hospitals as patients referred for specialist investigation and treatment	60
9	Lack of availability of Essential medicines	60
10	Cost of accessing healthcare (e.g., cost of accommodation and transport needed to receive treatment from centralized services)	60
11	Misdiagnosis of cancer at lower system levels (e.g., primary care, district hospital)	50
12	Lack of Diagnostic services (X-ray, Ultrasound, labs (e.g., PSA testing, biopsy facilities)	50
13	Lack of Radiotherapy options (brachytherapy/ teletherapy)	50
14	Preference for traditional, complementary, and alternative medicine	40
15	Lack of trust in healthcare and patients' citizens' rights (perceived quality; attitudes of healthcare workers; previous bad experience e.g., patients being turned away or refusal to refer; adequate consent)	40
16	Personal and professional obligations (financial and social implications to the patient and their families of seeking care and undergoing treatment)	40

For peer review only - http://bmjopen.bmj.com/site/about/guidelines.xhtml

17	Lack of social capital to support cancer journey especially where patients must travel for care (relationships, support from family, friends, colleagues)	40
18	Patient Fitness and treatment toxicity	40
19	Stigma associated with cancer diagnosis or severe illness/ Fears and beliefs around cancer	30
20	Communication/ language barrier between health care staff and patients	20
21	Staff motivation and burnout	20

In Round 2, four statements reached the consensus target (>70% of respondents scoring the statements >=15) as shown in table 3. These were: (i) Lack of diagnostic services (X-ray, Ultrasound, labs (e.g., PSA testing, biopsy facilities, (ii) Lack of availability of critical medicines, (iii) Lack of Radiotherapy options (brachytherapy/ teletherapy), and (iv) Cost of treatment e.g., surgery, radiotherapy, hormone therapy. In total, six research priorities were identified during the two round Delphi technique.

Table 3: Round 2 ranking

**Rank**	**Barrier**	**Ranking (% of participants scoring reason >=15)**
1	Lack of Diagnostic services (X-ray, Ultrasound, labs (e.g., PSA testing, biopsy facilities)	92
2	Lack of availability of critical medicines	92
3	Lack of Radiotherapy options (brachytherapy/ teletherapy)	83
4	Cost of treatment e.g., surgery, radiotherapy, hormone therapy	83
5	Lack of critical Surgical Supplies	67
6	Cost of accessing healthcare (e.g., cost of accommodation and transport needed to receive treatment from centralized services)	67
7	Accessibility of care (long distance/travel times to access specialist services)	58

For peer review only - http://bmjopen.bmj.com/site/about/guidelines.xhtml

8	Cost of diagnostic investigations	58
9	Misdiagnosis of cancer at lower system levels (e.g., primary care, district hospital)	50
10	Patient Fitness and treatment toxicity	50
11	Difficulties with healthcare coordination between regions and hospitals as patients referred for specialist investigation and treatment	50
12	Lack of trust in healthcare system and patients' citizens' rights (perceived quality; attitudes of healthcare workers; previous bad experience e.g., patients being turned away or refusal to refer; adequate consent)	33
13	Lack of Workforce (basic numbers low)	33
14	Preference for traditional, complementary, and alternative medicines	25
15	Personal and professional obligations (financial and social implications to the patient and their families of seeking care and undergoing treatment)	25
16	Lack of social capital to support cancer journey especially where patients must travel for care (relationships, support from family, friends, colleagues)	17

Discussion

The purpose of this Delphi technique was to prioritise key areas for research in prostate cancer in the Ugandan context and will form the basis of recommendation for cancer control planning and research investments. The Delphi process sought to priorities those factors that had the greatest impact on achieving timely access to high quality care, could be investigated empirically and for which interventions could be developed and implemented.

Our study invited cancer experts, clinicians, and policy experts in and outside cancer management and care to evaluate the top research priorities for prostate cancer care in Uganda. Six key domains were identified: (i) lack of awareness of cancer as a disease and recognition of symptoms, (ii) poor healthcare literacy, (iii) lack of diagnostic services, (iv) lack of availability of essential medicines, (v) lack of radiotherapy facilities (vi) cost of treatment.

For peer review only - http://bmjopen.bmj.com/site/about/guidelines.xhtml

With respect to these six areas there remains a paucity of literature to date in the Ugandan context but are important issues as outlined below.

Lack of awareness of cancer as a disease and poor recognition of cancer symptoms

Poor knowledge and misconceptions regarding prostate cancer and screening among Ugandan men,[24] is likely to contribute to diagnosis with advanced clinical stages (stage III and IV) of PC.[25] A lack of awareness about cancer and low health literacy has been reported as one of the major reasons for delays in seeking quality cancer care in sub-Saharan Africa.[15] Similarly, the lack of awareness of prostate cancer its symptoms and its consequences, definitely affects the uptake of screening and seeking specialist help that provides an opportunity for early detection of prostate cancer. The lack of adequate information and counselling on prostate cancer has been associated with the considerably low uptake of prostate cancer screening among Ugandan men.[26] Developing appropriate interventions for improving awareness and knowledge about prostate cancer are critical in supporting early detection and treatment outcomes of men who develop prostate cancer.

Low health literacy (when, how and where to seek services)

Healthcare literacy is defined as the degree to which individuals have the capacity to obtain, process, and understand basic health information and services needed to make appropriate health decisions and to successfully navigate the health care system.[27, 28] Poor healthcare literacy limits access to care, interaction with health services providers and illness management as people with low healthcare literacy are more likely to have poorer use of health services and therefore experience poorer health outcomes.[29] A recent study among prostate cancer survivors in Uganda has shown that inconsistent information or complete lack of information are common experiences of the prostate cancer survivors can affect their health outcomes.[30 31] Patients with limited health literacy may have limited knowledge and understanding of health and when coupled with lack of established follow-up mechanisms and inadequate social care support services experienced by many cancer patients in Uganda, this reduces their autonomy in self-care and decision making.[6]

Lack of diagnostic services

A lack of basic imaging and pathological services results in delays in confirming a cancer diagnosis and completing staging to support management options. Such delays have a knock-on effect on the timely delivery of treatment leads to high mortality, morbidity, and low

For peer review only - http://bmjopen.bmj.com/site/about/guidelines.xhtml

quality of life.[25 32 33] Lack of diagnostic services has led to delayed cancer diagnosis being a common in the Uganda setting.[31] Additionally, where the service has been obtained the high costs of diagnostic investigations compounded by the poor social-economic status of the patients has resulted in enormous delays in seeking prostate cancer care among Ugandan men.[6]

Lack of availability of essential medicines

A recent review has reported major barriers in access to core cancer medicines worldwide, with high prices are major barrier including medicine included in the WHO essential medicines list.[34] Even relatively low cost medicines (compared to chemotherapy and molecularly targeted anti-cancer agents) such as Goserellin are largely unavailable to patients in the Ugandan setting due to recurrent stock-outs and majority being costly.[6] A recent recommendation has suggested increased repurposing of existing drugs such as Metformin, Valproic acid initially intended for other conditions to treat prostate cancer.[35]

Lack of Radiotherapy facilities

This is a core treatment for prostate cancer particularly in high-risk disease. However, radiotherapy is not commonly available in sub Saharan Africa with limited experience for radiotherapy and brachytherapy, insufficient infrastructure as well as limited trained personnel and training opportunities.[36] Its low provision in Uganda has made the provision of treatment unachievable for many cancer sufferers. It has been previously noted that Uganda needs more than 20 operational radiotherapy units in order to respond adequately to its population demands.[37] Currently the only radiotherapy machines in the country are all located at the UCI in the capital city Kampala. This has resulted in demand for services not being met (simply patients do not receive it), prolonged waiting times, compromises timing between the administration of radiation doses and eventually clinical and treatment outcomes. Even when potentially lifesaving radiotherapy treatment options are made available, there are challenges with the cost of treatment for a known cornerstone of curative therapy.

The cost of treatment (surgery, radiotherapy, hormone therapy)

The cost of PC care is critical as there is limited access and availability to safe and reliable services including chemotherapy for prostate cancer patients in Uganda. The government of Uganda provides generic anticancer medicines for patients at the UCI at subsidised costs

For peer review only - http://bmjopen.bmj.com/site/about/guidelines.xhtml

under the Universal Health Coverage (UHC) strategy to make available the WHO Essential anticancer Medicine List (EML).[38] For example, at the UCI, patients pay a subsidised fee of approximately $85 before accessing radiotherapy services.[6] In general, most cancer patients in Uganda are not able to afford cancer therapy out of pocket and yet the UHC arrangement does not make available newer anticancer therapies. The proportion of anticancer agents especially the newer targeted therapies on the 2019 WHO EML that are available on the 2016 Uganda National Essential Medicines List (NEML) was about 70.5%. Essential medicines for prostate cancer including Leuprolide, Bicalutamide and Abiraterone are often out of stock through the government UHC strategy.[39] Availability and prices of anticancer agents vary widely in the LMICs including Uganda. The low availability and unaffordability of anticancer agents often lead patients to turn to alternative care approaches.[40] PC is among the commonest cancers treated with complimentary therapies in Uganda. The unavailability of critical medicines and curative treatments being out of reach has pushed many patients to seek alternative, traditional, Chinese, and complementary medicines chiefly from potential anticancer medicinal plants.[6 41]

Strengths and limitations

The Delphi technique was conducted by a multidisciplinary team following the CREDES guidelines.[16] The statements explored in the Delphi were identified through a systematic review. We were able to use both electronic web-based as well as face-to-face paper-based approaches to data collection. This study has some limitations: the response rates in round 1 and round 2 were relatively low (34% and 29%, respectively). However, the aim of this study was to have suitable representation to identify the top research priorities for prostate cancer care in Uganda rather than receiving a high response rate. As the survey was only completed by clinical and health system research experts, majority of whom were not familiar with the Delphi technique, how it works, and this may have resulted in bias. Further research is required to elicit patients' views. Although our questionnaires were sent to various groups and healthcare professionals from different clinical roles, most of the participants and experts in this study were oncologists and medical doctors. Future studies may need to consider other members of the healthcare team who were under-represented and may have alternative perspectives.

Conclusion

For peer review only - http://bmjopen.bmj.com/site/about/guidelines.xhtml

This study has identified the top six research priorities for prostate cancer in Uganda. Our findings have implications for designing appropriate and contextual prostate care services locally to ensure men have fewer barriers to receiving earlier diagnosis and high-quality affordable treatment and survivorship care.

Acknowledgements:

We thank the participants who participated in the Delphi technique and research team in Uganda where the study was conducted.

Author contributions:

AA conceived the study idea and designed it with AS, ADM and JS. AA, AS, AM, JS were engaged in the preparation and conduct of the Delphi study. AS, RM, DL, MM, SC, and AA led the writing of the paper and participated in the analysis. AA, ADM, and JS supervised the overall study. All authors read and approved the final manuscript.

Declaration of conflicting interests:

The author(s) declared no potential conflicts of interest with respect to the research, authorship, and/or publication of this article.

Patient and public involvement:

Patients and/or the public were not involved in the design, or conduct, or reporting, or dissemination plans of this research.

Patient consent for publication:

Not required.

Ethics approval:

Ethical approval was obtained from the Uganda Virus Research Institute Research Ethics Committee (UVRI-REC) Ref: GC/127/21/09/859 and the Uganda National Council for Science and Technology (UNCST) Ref: HS1790ES and the London School of Hygiene and Tropical Medicine Ref: 26672.

Funding:

For peer review only

The study was supported by funding from Wellcome's Institutional Strategic Support Fund grant no. 204928/Z/16/Z through the London School of Hygiene and Tropical Medicine.

Data sharing statement:

Please contact the corresponding author in regard to any data sharing requests. Please note there are ethical restrictions on sharing of data.

For peer review only

References

1. Sung H, Ferlay J, Siegel RL, et al. Global Cancer Statistics 2020: GLOBOCAN Estimates of Incidence and Mortality Worldwide for 36 Cancers in 185 Countries. *CA Cancer J Clin* 2021;71(3):209-49. doi: 10.3322/caac.21660 [published Online First: 2021/02/05]
2. Fitzmaurice C, Abate D, Abbasi N, et al. Global, Regional, and National Cancer Incidence, Mortality, Years of Life Lost, Years Lived With Disability, and Disability-Adjusted Life-Years for 29 Cancer Groups, 1990 to 2017: A Systematic Analysis for the Global Burden of Disease Study. *JAMA Oncol* 2019;5(12):1749-68. doi: 10.1001/jamaoncol.2019.2996 [published Online First: 2019/09/29]
3. Taitt HE. Global Trends and Prostate Cancer: A Review of Incidence, Detection, and Mortality as Influenced by Race, Ethnicity, and Geographic Location. *Am J Mens Health* 2018;12(6):1807-23. doi: 10.1177/1557988318798279 [published Online First: 2018/09/12]
4. Adeloye D, David RA, Aderemi AV, et al. An Estimate of the Incidence of Prostate Cancer in Africa: A Systematic Review and Meta-Analysis. *PLoS One* 2016;11(4):e0153496. doi: 10.1371/journal.pone.0153496 [published Online First: 2016/04/14]
5. Cassell A, Yunusa B, Jalloh M, et al. A Review of Localized Prostate Cancer: An African Perspective. *World J Oncol* 2019;10(4-5):162-68. doi: 10.14740/wjon1221 [published Online First: 2019/10/23]
6. Nakaganda A, Solt K, Kwagonza L, et al. Challenges faced by cancer patients in Uganda: Implications for health systems strengthening in resource limited settings. *J Cancer Policy* 2021;27:100263. doi: 10.1016/j.jcpo.2020.100263 [published Online First: 2021/03/01]
7. Singh K, Abdel Goad EH, Ramklass SS. Waiting times for prostate cancer diagnosis in KwaZulu-Natal, South Africa. *S Afr Med J* 2015;105(6):484-6. doi: 10.7196/samj.9192 [published Online First: 2015/12/31]

For peer review only - http://bmjopen.bmj.com/site/about/guidelines.xhtml

8. Omisanjo OA, Ogunremi OO, Akinola OO, et al. Waiting Times for Prostate Cancer Diagnosis in a Nigerian Population. *J Cancer Epidemiol* 2021;2021:5534683. doi: 10.1155/2021/5534683 [published Online First: 2021/08/28]
9. McGinley KF, Tay KJ, Moul JW. Prostate cancer in men of African origin. *Nat Rev Urol* 2016;13(2):99-107. doi: 10.1038/nrurol.2015.298 [published Online First: 2016/01/01]
10. Moodley J, Constant D, Mwaka AD, et al. Mapping awareness of breast and cervical cancer risk factors, symptoms and lay beliefs in Uganda and South Africa. *PloS one* 2020;15(10):e0240788. doi: 10.1371/journal.pone.0240788
11. Ilaboya D, Gibson L, Musoke D. Perceived barriers to early detection of breast cancer in Wakiso District, Uganda using a socioecological approach. *Global Health* 2018;14(1):9. doi: 10.1186/s12992-018-0326-0 [published Online First: 2018/01/25]
12. Ndejjo R, Mukama T, Kiguli J, et al. Knowledge, facilitators and barriers to cervical cancer screening among women in Uganda: a qualitative study. *BMJ Open* 2017;7(6):e016282. doi: 10.1136/bmjopen-2017-016282 [published Online First: 2017/06/14]
13. Wabinga HR, Nambooze S, Amulen PM, et al. Trends in the incidence of cancer in Kampala, Uganda 1991–2010. *International journal of cancer* 2014;135(2):432-39. doi: 10.1002/ijc.28661
14. Parkin DM, Nambooze S, Wabwire-Mangen F, et al. Changing cancer incidence in Kampala, Uganda, 1991-2006. *International journal of cancer* 2010;126(5):1187-95. doi: 10.1002/ijc.24838 [published Online First: 2009/08/19]
15. Lombe DC, Mwamba M, Msadabwe S, et al. Delays in seeking, reaching and access to quality cancer care in sub-Saharan Africa: a systematic review. *BMJ Open* 2023;13(4):e067715. doi: 10.1136/bmjopen-2022-067715 [published Online First: 2023/04/14]
16. Jünger S, Payne SA, Brine J, et al. Guidance on Conducting and REporting DElphi Studies (CREDES) in palliative care: Recommendations based on a methodological systematic review. *Palliat Med* 2017;31(8):684-706. doi: 10.1177/0269216317690685 [published Online First: 2017/02/14]
17. Jones J, Hunter D. Consensus methods for medical and health services research. *Bmj* 1995;311(7001):376-80. doi: 10.1136/bmj.311.7001.376 [published Online First: 1995/08/05]
18. Boulkedid R, Abdoul H, Loustau M, et al. Using and reporting the Delphi method for selecting healthcare quality indicators: a systematic review. *PLoS One* 2011;6(6):e20476. doi: 10.1371/journal.pone.0020476 [published Online First: 2011/06/23]
19. Cotache-Condor C, Kantety V, Grimm A, et al. Determinants of delayed childhood cancer care in low- and middle-income countries: A systematic review. *Pediatr Blood Cancer* 2023;70(3):e30175. doi: 10.1002/pbc.30175 [published Online First: 2022/12/30]
20. Pacagnella RC, Cecatti JG, Osis MJ, et al. The role of delays in severe maternal morbidity and mortality: expanding the conceptual framework. *Reprod Health Matters* 2012;20(39):155-63. doi: 10.1016/s0968-8080(12)39601-8 [published Online First: 2012/07/14]
21. Thaddeus S, Maine D. Too far to walk: maternal mortality in context. *Soc Sci Med* 1994;38(8):1091-110. doi: 10.1016/0277-9536(94)90226-7 [published Online First: 1994/04/01]
22. Schneider P, Evaniew N, Rendon JS, et al. Moving forward through consensus: protocol for a modified Delphi approach to determine the top research priorities in the field of orthopaedic oncology. *BMJ Open* 2016;6(5):e011780. doi: 10.1136/bmjopen-2016-011780 [published Online First: 2016/05/26]
23. Harris PA, Taylor R, Minor BL, et al. The REDCap consortium: Building an international community of software platform partners. *J Biomed Inform* 2019;95:103208. doi: 10.1016/j.jbi.2019.103208 [published Online First: 2019/05/13]
24. Nakandi H, Kirabo M, Semugabo C, et al. Knowledge, attitudes and practices of Ugandan men regarding prostate cancer. *Afr J Urol* 2013;19(4):165-70. doi: 10.1016/j.afju.2013.08.001 [published Online First: 2014/09/16]

For peer review only - http://bmjopen.bmj.com/site/about/guidelines.xhtml

25. Yahaya JJ, Okecha T, Odida M, et al. Prognostic Factors for Overall Survival of Patients with Prostate Cancer in Kyadondo County, Uganda. *Prostate Cancer* 2020;2020:8517130. doi: 10.1155/2020/8517130 [published Online First: 2020/05/16]
26. Ekwan R, Bua E, Nantale R, et al. Uptake of prostate cancer screening and associated factors among men aged 50 years and above in Lira city, Uganda: a cross-sectional study. *BMC Public Health* 2023;23(1):432. doi: 10.1186/s12889-023-15348-w [published Online First: 2023/03/07]
27. Hersh L, Salzman B, Snyderman D. Health Literacy in Primary Care Practice. *Am Fam Physician* 2015;92(2):118-24. [published Online First: 2015/07/16]
28. Samerski S. Health literacy as a social practice: Social and empirical dimensions of knowledge on health and healthcare. *Soc Sci Med* 2019;226:1-8. doi: 10.1016/j.socscimed.2019.02.024 [published Online First: 2019/03/05]
29. Budhathoki SS, Pokharel PK, Good S, et al. The potential of health literacy to address the health related UN sustainable development goal 3 (SDG3) in Nepal: a rapid review. *BMC Health Serv Res* 2017;17(1):237. doi: 10.1186/s12913-017-2183-6 [published Online First: 2017/03/30]
30. Nabisubi P, Nanyingi M, Okeny PK. Lived experiences of prostate cancer patients below 55 years of age: A phenomenological study of outpatients receiving treatment at the Uganda Cancer Institute. *East and Central African Journal of Surgery,* 2020 doi: https://doi.org/10.4314/ecajs.v25i3.3
31. Bunani N, Kisakye AN, Ssennyonjo A, et al. Late Diagnosis among patients with Prostate Cancer at the Uganda Cancer Institute: A retrospective cohort study. *Research Square* 2020 doi: https://doi.org/10.21203/rs.2.11766/v3
32. Seraphin TP, Joko-Fru WY, Manraj SS, et al. Prostate cancer survival in sub-Saharan Africa by age, stage at diagnosis, and human development index: a population-based registry study. *Cancer Causes Control* 2021;32(9):1001-19. doi: 10.1007/s10552-021-01453-x [published Online First: 2021/07/11]
33. Hanna TP, King WD, Thibodeau S, et al. Mortality due to cancer treatment delay: systematic review and meta-analysis. *Bmj* 2020;371:m4087. doi: 10.1136/bmj.m4087 [published Online First: 2020/11/06]
34. Fundytus A, Sengar M, Lombe D, et al. Access to cancer medicines deemed essential by oncologists in 82 countries: an international, cross-sectional survey. *Lancet Oncol* 2021;22(10):1367-77. doi: 10.1016/s1470-2045(21)00463-0 [published Online First: 2021/09/25]
35. Bahmad HF, Demus T, Moubarak MM, et al. Overcoming Drug Resistance in Advanced Prostate Cancer by Drug Repurposing. *Med Sci (Basel)* 2022;10(1) doi: 10.3390/medsci10010015 [published Online First: 2022/03/01]
36. Harvey N, Adeyoju A, Brough R. Prostate Cancer in Sub-Saharan Africa: Diagnosis and Management. Cancer in Sub-Saharan Africa: Springer 2017:95-107.
37. Massoud S. Challenges of making radiotherapy accessible in developing countries. *Cancer Control* 2013;2013:85-94.
38. WHO. World Health Organization Model List of Essential Medicines, 21st List, 2019. Geneva: World

Health Organization; 2019. Licence: CC BY-NC-SA 3.0 IGO. *Available at: https://appswhoint/iris/bitstream/handle/10665/325771/WHO-MVP-EMP-IAU-201906-engpdf?sequence=1&isAllowed=y Last accessed on 9th May 2023* 2019
39. Kizub DA, Naik S, Abogan AA, et al. Access to and Affordability of World Health Organization Essential Medicines for Cancer in Sub-Saharan Africa: Examples from Kenya, Rwanda, and Uganda. *The oncologist* 2022;27(11):958-70. doi: 10.1093/oncolo/oyac143
40. Ocran Mattila P, Ahmad R, Hasan SS, et al. Availability, Affordability, Access, and Pricing of Anti-cancer Medicines in Low- and Middle-Income Countries: A Systematic Review of Literature. *Frontiers in Public Health* 2021;9 doi: 10.3389/fpubh.2021.628744
41. Omara T, Kiprop AK, Ramkat RC, et al. Medicinal Plants Used in Traditional Management of Cancer in Uganda: A Review of Ethnobotanical Surveys, Phytochemistry, and Anticancer Studies. *Evid*

For peer review only - http://bmjopen.bmj.com/site/about/guidelines.xhtml

Based Complement Alternat Med 2020;2020:3529081. doi: 10.1155/2020/3529081 [published Online First: 2020/04/08]

For peer review only

Supplemental Appendix 1

Delphi process round 1 Questionnaire.docx

Supplemental Appendix 2

Delphi process round 2 Questionnaire.docx

Guidance Document for Completion of the APC-DOUG Survey

The guidance below relates to Round 1 of the Delphi process.

The aim of Round 1 is to prioritize for further research and intervention development, the major barriers that exist in accessing prostate cancer care. We have considered three aspects to the delays that may occur in receiving a diagnosis and/or treatment.

Delay 1 (seeking care): This is the delay in recognizing illness and deciding to seek appropriate medical help outside the home.

Delay 2 (reaching care): This is the delay in reaching an appropriate health facility.

***Delay 3 (receiving quality care): This is the delay in receiving quality care after reaching the health facility*.**

We are kindly asking you to review and answer the questions below based on your experience.

Instructions:

1. There are 21 statements identified from a review of the wider empirical literature as a reason for delay in accessing cancer care.
2. We want to understand how important or relevant these reasons for delays are with respect to prostate cancer in Uganda
3. Each statement will be scored out of 20 based on 4 assessment domains a. feasibility to study this issue; b. scale of the issue; c. impact of the barrier on prostate cancer outcomes; d. feasibility of addressing this barrier
4. Please score each domain on each delay out of 5, with 5 being the highest (most important) and 1 being the least (important).
5. The statements assigned the highest scores will be considered as main priorities for round 2 of the Delphi process.

If you have any questions, please contact Dr Amos D. Mwaka email: mwakaad@yahoo.com or Andrew S. Ssemata email: andrew.ssemata@mrcuganda.org.

For peer review only - http://bmjopen.bmj.com/site/about/guidelines.xhtml

Reason for delay	Feasible (Easy to measure or investigate this reason for delay)	Large scale (This delay affects a significant proportion of prostate cancer patients)	High Impact (This delay is a significant or avoidable cause of death or disability from prostate cancer)	Modifiable (The reason identified can be readily changed to improve care)
	Please score each theme for each statement with a score of 1 – 5 based on the explanation in brackets (1 is lowest and 5 is highest)			
1. Accessibility of care (long distance/travel times to access specialist services)				
2. Lack of awareness of cancer as a disease and recognition of symptoms				
3. Poor healthcare literacy (when, how and where to seek services)				
4. Preference for traditional, complementary, and alternative medicines				
5. Lack of trust in healthcare system and patients' citizens' rights (perceived quality; attitudes of healthcare workers; previous bad experience e.g., patients being turned away or refusal to refer; adequate consent)				
6. Personal and professional obligations (financial and social implications to the patient and their families of seeking care and undergoing treatment)				
7. Stigma associated with a cancer diagnosis or severe illness/ Fears and beliefs around cancer				
8. Lack of social capital (relationships, support from family, friends, colleagues)				

For peer review only - http://bmjopen.bmj.com/site/about/guidelines.xhtml

For peer review only

Reason for delay	Feasible (Easy to measure or investigate this reason for delay)	Large scale (This delay affects a significant proportion of prostate cancer patients)	High Impact (This delay is a significant or avoidable cause of death or disability from prostate cancer)	Modifiable (The reason identified can be readily changed to improve care)
9. Misdiagnosis of cancer (at lower system levels e.g. primary care, district hospital)				
10. Difficulties with healthcare coordination between regions and hospitals as patients referred for specialist investigation and treatment				
11. Lack of diagnostic services (X-ray, Ultrasound, labs (e.g. PSA testing, biopsy facilities)				
12. Lack of availability of critical medicines				
13. Lack of radiotherapy options (brachytherapy/ teletherapy)				
14. Lack of critical surgical supplies				
15. Cost of accessing healthcare (e.g., cost of accommodation and transport needed to receive treatment from centralized services)				
16. Cost of diagnostic investigations				
17. Cost of treatments e.g. surgery, radiotherapy. Hormone therapy				
18. Lack of workforce (basic numbers low and inadequately trained staff)				
19. Patient fitness and treatment toxicity				
20. Communication/language barriers between health care staff and patients				
21. Staff motivation and burnout				

For peer review only - http://bmjopen.bmj.com/site/about/guidelines.xhtml

For peer review only

For peer review only

For peer review only - http://bmjopen.bmj.com/site/about/guidelines.xhtml

Guidance Document for Completion of the APC-DOUG Survey

The guidance below relates to Round 2 of the Delphi process.

The aim of Round 2 is to prioritize for further research and intervention development, the major barriers that exist in accessing prostate cancer care. We have considered three aspects to the delays that may occur in receiving a diagnosis and/or treatment.

Delay 1 (seeking care): This is the delay in recognizing illness and deciding to seek appropriate medical help outside the home.

Delay 2 (reaching care): This is the delay in reaching an appropriate health facility.

Delay 3 (receiving quality care): This is the delay in receiving quality care after reaching the health facility.

We are kindly asking you to review and answer the questions below based on your experience.

Instructions:

1. There are 18 statements identified from a review of the wider empirical literature as a reason for delay in accessing cancer care.
2. We want to understand how important or relevant these reasons for delays are with respect to prostate cancer in Uganda
3. Each statement will be scored out of 20 based on 4 assessment domains a. feasibility to study this issue; b. scale of the issue; c. impact of the barrier on prostate cancer outcomes; d. feasibility of addressing this barrier
4. Please score each domain on each delay out of 5, with 5 being the highest (most important) and 1 being the least (important).
5. The statements assigned the highest scores will be considered as main priorities for round 2 of the Delphi process.

If you have any questions, please contact Dr Amos D. Mwaka email: mwakaad@yahoo.com or Andrew S. Ssemata email: andrew.ssemata@mrcuganda.org.

For peer review only - http://bmjopen.bmj.com/site/about/guidelines.xhtml

Reason for delay	Feasible (Easy to measure or investigate this reason for delay)	Large scale (This delay affects a significant proportion of prostate cancer patients)	High Impact (This delay is a significant or avoidable cause of death or disability from prostate cancer)	Modifiable (The reason identified can be readily changed to improve care)
	Please score each theme for each statement with a score of 1 – 5 based on the explanation in brackets (1 is lowest and 5 is highest)			
1. Accessibility of care (long distance/travel times to access specialist services)				
2. Lack of awareness of cancer as a disease and recognition of symptoms				
3. Poor healthcare literacy (when, how and where to seek services)				
4. Preference for traditional, complementary, and alternative medicines				
5. Lack of trust in healthcare system and patients' citizens' rights (perceived quality; attitudes of healthcare workers; previous bad experience e.g., patients being turned away or refusal to refer; adequate consent)				
6. Personal and professional obligations (financial and social implications to the patient and their families of seeking care and undergoing treatment)				
7. Lack of social capital (relationships, support from family, friends, colleagues)				

For peer review only - http://bmjopen.bmj.com/site/about/guidelines.xhtml
For peer review only

Reason for delay	Feasible (Easy to measure or investigate this reason for delay)	Large scale (This delay affects a significant proportion of prostate cancer patients)	High Impact (This delay is a significant or avoidable cause of death or disability from prostate cancer)	Modifiable (The reason identified can be readily changed to improve care)
8. Misdiagnosis of cancer (at lower system levels e.g. primary care, district hospital)				
9. Difficulties with healthcare coordination between regions and hospitals as patients referred for specialist investigation and treatment				
10. Lack of diagnostic services (X-ray, Ultrasound, labs (e.g. PSA testing, biopsy facilities)				
11. Lack of availability of critical medicines				
12. Lack of radiotherapy options (brachytherapy/ teletherapy)				
13. Lack of critical surgical supplies				
14. Cost of accessing healthcare (e.g., cost of accommodation and transport needed to receive treatment from centralized services)				
15. Cost of diagnostic investigations				
16. Cost of treatments e.g. surgery, radiotherapy. Hormone therapy				
17. Lack of workforce (basic numbers low and inadequately trained staff)				
18. Patient fitness and treatment toxicity				

For peer review only - http://bmjopen.bmj.com/site/about/guidelines.xhtml

Reporting checklist for cross sectional study.

Based on the STROBE cross sectional guidelines.

Instructions to authors

Complete this checklist by entering the page numbers from your manuscript where readers will find each of the items listed below.

Your article may not currently address all the items on the checklist. Please modify your text to include the missing information. If you are certain that an item does not apply, please write "n/a" and provide a short explanation.

Upload your completed checklist as an extra file when you submit to a journal.

In your methods section, say that you used the STROBE cross sectionalreporting guidelines, and cite them as:

von Elm E, Altman DG, Egger M, Pocock SJ, Gotzsche PC, Vandenbroucke JP. The Strengthening the Reporting of Observational Studies in Epidemiology (STROBE) Statement: guidelines for reporting observational studies.

		Reporting Item	Page Number
**Title and abstract**			
Title	#1a	Indicate the study's design with a commonly used term in the title or the abstract	1
Abstract	#1b	Provide in the abstract an informative and balanced summary of what was done and what was found	2
**Introduction**			
Background / rationale	#2	Explain the scientific background and rationale for the investigation being reported	3-4
Objectives	#3	State specific objectives, including any prespecified hypotheses	4
**Methods**			
Study design	#4	Present key elements of study design early in the paper	4
Setting	#5	Describe the setting, locations, and relevant dates, including periods	5

For peer review only

		of recruitment, exposure, follow-up, and data collection	
Eligibility criteria	#6a	Give the eligibility criteria, and the sources and methods of selection of participants.	6
	#7	Clearly define all outcomes, exposures, predictors, potential confounders, and effect modifiers. Give diagnostic criteria, if applicable	6
Data sources / measurement	#8	For each variable of interest give sources of data and details of methods of assessment (measurement). Describe comparability of assessment methods if there is more than one group. Give information separately for for exposed and unexposed groups if applicable.	6
Bias	#9	Describe any efforts to address potential sources of bias	6
Study size	#10	Explain how the study size was arrived at	N/A purposive sample
Quantitative variables	#11	Explain how quantitative variables were handled in the analyses. If applicable, describe which groupings were chosen, and why	6
Statistical methods	#12a	Describe all statistical methods, including those used to control for confounding	6
Statistical methods	#12b	Describe any methods used to examine subgroups and interactions	N/A
Statistical methods	#12c	Explain how missing data were addressed	N/A
Statistical methods	#12d	If applicable, describe analytical methods taking account of sampling strategy	N/A
Statistical methods	#12e	Describe any sensitivity analyses	N/A
**Results**			
Participants	#13a	Report numbers of individuals at each stage of study—eg numbers potentially eligible, examined for eligibility, confirmed eligible, included in the study, completing follow-up, and analysed. Give information separately for for exposed and unexposed groups if	6

For peer review only

		applicable.	
Participants	#13b	Give reasons for non-participation at each stage	7
Participants	#13c	Consider use of a flow diagram	N/A
Descriptive data	#14a	Give characteristics of study participants (eg demographic, clinical, social) and information on exposures and potential confounders. Give information separately for exposed and unexposed groups if applicable.	7-8
Descriptive data	#14b	Indicate number of participants with missing data for each variable of interest	N/A
Outcome data	#15	Report numbers of outcome events or summary measures. Give information separately for exposed and unexposed groups if applicable.	N/A
Main results	#16a	Give unadjusted estimates and, if applicable, confounder-adjusted estimates and their precision (eg, 95% confidence interval). Make clear which confounders were adjusted for and why they were included	N/A
Main results	#16b	Report category boundaries when continuous variables were categorized	7-11
Main results	#16c	If relevant, consider translating estimates of relative risk into absolute risk for a meaningful time period	7-11
Other analyses	#17	Report other analyses done—e.g., analyses of subgroups and interactions, and sensitivity analyses	N/A
**Discussion**			
Key results	#18	Summarise key results with reference to study objectives	11
Limitations	#19	Discuss limitations of the study, taking into account sources of potential bias or imprecision. Discuss both direction and magnitude of any potential bias.	14
Interpretation	#20	Give a cautious overall interpretation considering objectives, limitations, multiplicity of analyses, results from similar studies, and other relevant evidence.	14
Generalisability	#21	Discuss the generalisability (external validity) of the study results	14

For peer review only

Other Information

Funding	#22	Give the source of funding and the role of the funders for the present study and, if applicable, for the original study on which the present article is based	15

Notes:

- 10: N/A purposive sample The STROBE checklist is distributed under the terms of the Creative Commons Attribution License CC-BY. This checklist was completed on 17. May 2023 using https://www.goodreports.org/, a tool made by the EQUATOR Network in collaboration with Penelope.ai

For peer review only

BMJ Open

Moving forward through consensus: a national Delphi approach to determine the top research priorities in prostate cancer in Uganda.

Journal:	*BMJ Open*
Manuscript ID	bmjopen-2023-075739.R1
Article Type:	Original research
Date Submitted by the Author:	01-Nov-2023
Complete List of Authors:	Ssemata, Andrew; MRC/UVRI and LSHTM Uganda Research Unit, Social Aspects of Health Across the Lifecourse; London School of Hygiene & Tropical Medicine, Department of Global Health and Development<br>Muhumuza, Richard; MRC/UVRI & LSHTM Uganda Research Unit, Social Aspects of Health across the Lifecourse<br>Seeley, Janet; MRC/UVRI and LSHTM Uganda Research Unit, Social Aspects of Health across the Lifecourse; London School of Hygiene and Tropical Medicine, Department of Global Health and Development<br>Lombe, Dorothy; MidCentral District Health Board, Radiation Oncology<br>Mwamba, Monde; University of Zambia<br>Msadabwe, Susan; Cancer Diseases Hospital<br>Mwaka, Amos; Mulago Hospital/Makerere University, Department of Internal Medicine; Gulu University, Department of Medicine, Faculty of Medicine<br>Aggarwal, Ajay; London School of Hygiene and Tropical Medicine Faculty of Public Health and Policy, Health Services Research & Policy; King's College London
**Primary Subject Heading**:	Oncology
Secondary Subject Heading:	Health policy, Health services research, Global health, Oncology
Keywords:	Prostate, Primary Health Care, Prostate disease < UROLOGY, Quality in health care < HEALTH SERVICES ADMINISTRATION & MANAGEMENT, Health Services, ONCOLOGY

SCHOLARONE™
Manuscripts

I, the Submitting Author has the right to grant and does grant on behalf of all authors of the Work (as defined in the below author licence), an exclusive licence and/or a non-exclusive licence for contributions from authors who are: i) UK Crown employees; ii) where BMJ has agreed a CC-BY licence shall apply, and/or iii) in accordance with the terms applicable for US Federal Government officers or employees acting as part of their official duties; on a worldwide, perpetual, irrevocable, royalty-free basis to BMJ Publishing Group Ltd ("BMJ") its licensees and where the relevant Journal is co-owned by BMJ to the co-owners of the Journal, to publish the Work in this journal and any other BMJ products and to exploit all rights, as set out in our licence.

The Submitting Author accepts and understands that any supply made under these terms is made by BMJ to the Submitting Author unless you are acting as an employee on behalf of your employer or a postgraduate student of an affiliated institution which is paying any applicable article publishing charge ("APC") for Open Access articles. Where the Submitting Author wishes to make the Work available on an Open Access basis (and intends to pay the relevant APC), the terms of reuse of such Open Access shall be governed by a Creative Commons licence – details of these licences and which Creative Commons licence will apply to this Work are set out in our licence referred to above.

Other than as permitted in any relevant BMJ Author's Self Archiving Policies, I confirm this Work has not been accepted for publication elsewhere, is not being considered for publication elsewhere and does not duplicate material already published. I confirm all authors consent to publication of this Work and authorise the granting of this licence.

For peer review only

Word count: 4175

Moving forward through consensus: a national Delphi approach to determine the top research priorities in prostate cancer in Uganda.

Authors:

Andrew Sentoogo Ssemata[1, 2*], Richard Muhumuza[1], Janet Seeley[1, 2], Dorothy Chilambe Lombe[3], Monde Mwamba[4], Susan Msadabwe[5], Amos Deogratius Mwaka[6,7], Ajay Aggarwal[8,9]

1. **Affiliations:** The Medical Research Council/ Uganda Virus Research Institute and London School of Hygiene & Tropical Medicine (MRC/UVRI & LSHTM) Uganda Research Unit, Entebbe, Uganda.
2. Department of Global Health & Development, London School of Hygiene and Tropical Medicine, London, UK.
3. Radiation Oncology, Te Whatu Ora Health NZ MidCentral, Palmerston North, New Zealand.
4. ZAMBART, Lusaka, Zambia.
5. Department of Radiation Oncology, Cancer Diseases Hospital, Lusaka, Zambia.
6. Department of Medicine, College of Health Sciences, Makerere University, Kampala, Uganda.
7. Department of Medicine, Faculty of Medicine, Gulu University, Gulu, Uganda
8. Department of Health Services Research and Policy, London School of Hygiene and Tropical Medicine, London, UK.
9. King's College London, London, UK.

***Correspondence to:** andrewssemata@yahoo.co.uk

For peer review only - http://bmjopen.bmj.com/site/about/guidelines.xhtml

Abstract

Objective: To identify key areas for research in prostate cancer (PC) in the Ugandan context by establishing the major health system, socioeconomic and clinical barriers to seeking, reaching, and receiving high quality cancer care.

Design: Modified Delphi Technique

Setting: Government and private-not-for-profit hospitals

Methods: We applied a two-stage modified Delphi technique to identify the consensus view across cancer experts. In Round 1, experts received a questionnaire containing 21 statements drawn from a systematic review identifying the reason for the delay in accessing cancer care. Each statement was scored out of 20. Statements scoring ≥15 from over 70% of participants were prioritized for inclusion while statements for which <30% of participants gave a score of ≥15 were excluded. Sixteen statements were included in Round 2 as they did not receive consensus for inclusion or exclusion.

Results: We found that the top six research priority areas arise from challenges including: (i) Lack of diagnostic services - Ultrasound, laboratory tests, and biopsy facilities; (ii) High costs of services e.g., surgery, radiotherapy, hormone therapy are unaffordable to most patients, (iii) Lack of essential medicines, (iv) Limited radiotherapy capacity (v) Lack of awareness of cancer as a disease and low recognition of symptoms, (vi) Low Healthcare literacy. The lack of critical surgical supplies, high diagnostic and treatment costs were ranked highest in order of importance in round one. Round two also revealed lack of diagnostic services, unavailability of critical medicines, lack of radiotherapy options, high costs of treatments and lack of critical surgical supplies as the top priorities.

Conclusion: These research priority areas ought to be addressed in future research to improve prompt prostate cancer diagnosis and care in Uganda. There is need to improve the supply of high-quality affordable anticancer medicines for prostate cancer patients so as to improve the survivorship from the cancer.

Keywords: Prostate cancer, delays, health care, Uganda, Delphi technique

Article Summary

Strengths and limitations of this study

For peer review only - http://bmjopen.bmj.com/site/about/guidelines.xhtml

- This is a novel Delphi study exploring research priorities for prostate cancer research in Uganda from various stakeholders in cancer care.
- The study was informed by a recent systematic review which provided insight into the statements explored in the Delphi technique.
- Participants were recruited from various clinical, government aided and private settings and geographical locations giving representativeness of the participants.
- A limitation to this study was the low response rates in round 1 and round 2 as most stakeholders were not familiar with the Delphi technique, or how it works.

For peer review only

Introduction

In 2020, there were 19.3 million new cancer cases and 10 million cancer deaths worldwide. (1) Prostate cancer is among the top 3 most common incident cancers in men (1.3 million incident cases) and the leading cause of cancer-related deaths for men in 56 countries including Uganda. (2)

A recent review has presented a worrying disparity in the mortality to incidence ratio from prostate cancer at 90% in Africa compared to 10% in North America. (3) For a curable cancer these figures are stark. In the low- and middle-income countries (LMICs), one of the challenges facing cancer control is the late diagnosis with advanced stage cancers at diagnosis. (4, 5, 6) The reasons that contribute to delayed diagnoses and advanced stage at presentation include delayed recognition of symptoms and signs of cancers by patients and primary healthcare professionals, inadequate health and cancer information for patients, poor continuity of care, lack of facilities for biopsy and histological diagnoses, costs and availability for treatments including drug therapies. (6, 7, 8)

One of the key challenges to delivering timely prostate cancer care is that the condition is largely asymptomatic in its early stages. African men also face much higher prostate cancer incidence, severity, and greater risk of death than is seen in Caucasian populations, leading to much greater economic impacts on societies. (9)

For peer review only - http://bmjopen.bmj.com/site/about/guidelines.xhtml

From a cancer control perspective, to date, in Uganda, there has been much more emphasis on cancers affecting women, especially breast and cervical cancers than those affecting men such as prostate cancer. (10, 11, 12) Little attention has been given to the cancers affecting men especially prostate cancer despite increasing in incidence over the recent decades. (13, 14, 15) There is need to inform national control plans and develop the necessary infrastructure to manage this growing cancer burden. , there is a need to prioritise key areas for research.

We present findings from the African Prostate Cancer - Disparities in Outcomes in Uganda (APC-DOUG) study whose aim was to describe health system, socioeconomic and clinical factors that influence access to and outcomes of care for prostate cancer across hospitals in Uganda. The purpose of this Delphi process was to prioritise key areas for research in prostate cancer in the Ugandan context by establishing the major barriers to seeking cancer care, reaching cancer care, and receiving high quality cancer care. By defining future research priorities and investigations, this will help to support the future development of health system interventions to reduce inequalities and improve prostate cancer outcomes in hospitals across Uganda.

Methods

The Delphi technique is a relevant source of evidence in health care research foundational in the formation of consensus or the exploration of a field beyond existing knowledge. (16) It has been used in medicine to develop research priorities. (16, 17, 18)

A structured 2-round modified Delphi technique with Ugandan clinical and health system research experts involved in prostate cancer care was conducted between December 2021 and July 2022. The Delphi technique was used to elicit consensus on the key research priorities in relation to the delays that may occur in receiving a diagnosis and/or treatment based on the Three Delays framework. (19, 20) The "Three Delays" Framework has been effective as a rapid health system assessment tool to understand complex multi-factorial problems affecting access to care. (20) The Three delays framework focused on delays to: (i) Seeking care - delays in recognizing illness and deciding to seek appropriate medical help outside the home;

For peer review only - http://bmjopen.bmj.com/site/about/guidelines.xhtml

(ii) Reaching care - delays in reaching an appropriate health facility and (iii) Receiving quality care - delays in receiving quality care after reaching the health facility. (21)

Development of the survey

The Delphi survey was informed by two systematic reviews undertaken by the study team appraising primary quantitative, (15) and qualitative (Mwamba et al., under peer review) research studies that had sought to identify the factors influencing delays in cancer diagnosis and treatment as well as receiving quality care in Sub-Saharan Africa. From this literature review 22 key factors contributing to delays were identified and represented priority areas for further investigation. These were translated into written statements that formed part of the Delphi consensus exercise for prioritizing research in prostate cancer in Uganda (supplemental appendix 1).

Data collection

Data collection for round 1 of the Delphi process was conducted between December 2021 and February 2022 and round 2 of the Delphi process between March and July 2022. Participants were selected to ensure representation from different regions, health care sectors (primary and secondary); specialist disciplines (urology, oncology); academia and public health as well as patient representatives. Lists of participants to approach from both public and private health facilities in Kampala, Wakiso, and Mukono districts were developed by the planning committee which comprised two oncologists, and two health services research methodological experts who oversaw the design, execution, and analysis of all phases of the study.

At present there is only one major comprehensive cancer treatment centre in Uganda (the Uganda Cancer Institute, based at Mulago Hospital) with two active regional bases (in Mbarara, western Uganda, and Gulu; Northern Uganda), (6) and representation from each of these were sought. A snowballing approach was also undertaken where participants recommended additional stakeholders for participation. Agreement was reached regarding participant selection, consensus thresholds, and survey format and question structure. For round 1, we approached participants from the Uganda Cancer Institute, government hospitals, Private-for-profit hospitals, Private-not-for-profit hospitals, Ministry of Health, and a university.

For peer review only - http://bmjopen.bmj.com/site/about/guidelines.xhtml

Due to low response rates (either non-response or incomplete questionnaires received from round 1) despite phone and face-to-face follow with the participants to ensure completion it was necessary to invite an additional set of participants. It was decided by the research team to broaden the profile of participants to include cancer experts, clinicians, policy experts involved in cancer management from centres that provide cancer care services and work in liaison with the specialist centres included in round 1 to participate in round 2 of the Delphi process.

Procedure

Experienced social science and clinical researchers conducted the data collection using a survey questionnaire. Participants were approached by a formal invitation to participate by email correspondence followed by a phone call and for some participants a face-to-face meeting. For those willing to participate, we collected demographic data, and a structured questionnaire was hand delivered to the participants. This was followed up with a reminder phone call within a four-week period to ensure completion of the question. Two sequential rounds of questionnaires were used for this Delphi process (Supplemental appendix 1 and 2).

Round 1 consisted of scoring the 22 statements using a 4-point scale. Each statement was assigned a score (out of 20) based on the four criteria, each scored out of 5, as below:

- **Feasible** – how easy is it to measure or assess this reason for the delay?
- **Large scale** – does this factor affect a considerable proportion of men with prostate cancer.
- **High Impact** –is this factor a significant cause of death or disability from prostate cancer?
- **Modifiable** – can this factor be reasonably addressed to improve the care of men with prostate cancer.

Once all the questionnaires for round 1 were received, we compiled the rankings for each statement. Based on the criteria by Schneider et al, (22) statements were selected as follows. Statements where 70% of participants gave a score of ≥15 were included as research priorities. Statements for which <30% of participants gave a score of ≥15 were excluded at this stage. (22) The rest of the statements went forward to the second round of the Delphi consensus process. The information provided in the round 1 of the Delphi technique was

For peer review only - http://bmjopen.bmj.com/site/about/guidelines.xhtml

collated and summarized in REDCap software, (23) to enable formal analysis, and to formulate a second questionnaire with fewer statements based on the selection criteria above.

Round 2 of the Delphi included 18 statements. An electronic questionnaire designed in REDCap was generated and emailed to 42 participants. Participants were followed up with a reminder phone call within a four-week period and through face-to-face meetings by the study team. Recurrent emails and phone calls were made to those who had been contacted and had not completed the survey.

Ethical considerations

This study was performed in line with the principles of the Declaration of Helsinki. Ethical approval to conduct the Delphi technique was obtained from the Uganda Virus Research Institute Research Ethics Committee (UVRI-REC) Ref: GC/127/21/09/859 and the Uganda National Council for Science and Technology (UNCST) Ref: HS1790ES and the London School of Hygiene and Tropical Medicine Ref: 26672. Each participant provided written informed consent before any study procedures were conducted and were reimbursed for their time and participation in the study. Each participant was provided a time compensation of 30,000 Uganda shillings at the end of each completed questionnaire.

Patient and public involvement

Patients were not involved in the design, or conduct, or reporting, or dissemination plans of this research.

Results

Ten and twelve respondents completed the survey questionnaires in round 1 and 2 respectively. In round 1, a total of 30 clinicians were invited through email and face to face meetings, of which 10 (34%) participated and completed the survey. The majority of the participants in this round were oncologists $n = 4$ (40%) and cancer researchers $n = 3$ (30%). Most of the participants worked in a cancer care facility. In round 2, a total of 42 clinicians were invited through email, online and face to face meetings, of which 12 (29%) participated and completed the survey. Most of the participants in this round were radiologists $n = 3$ (25%) and general practitioners $n = 3$ (25%). Most of the participants in round 2 operated from a general health care setting. Of the 22 people who participated in round 1 and 2, three

For peer review only - http://bmjopen.bmj.com/site/about/guidelines.xhtml

For peer review only

participated in both rounds and seven participants participated in round 1 only. The characteristics of the respondents in Rounds 1 and 2 are presented in table 1.

Table 1 showing characteristics of participants in round 1 and 2 of the Delphi process.

	**ROUND 1**		
	**Gender**	**Profession**	**Organisation**
1	Male	Oncologist	Cancer Institute, Kampala
2	Male	Oncologist	Cancer Institute, Kampala
3	Male	Oncologist	Cancer Institute, Kampala
4	Male	Cancer researcher and administrator	Cancer Institute, Kampala
5	Male	Oncologist	Cancer Institute, Kampala
6	Male	Public health specialist and policy expert	University/ Ministry of Health, Kampala
7	Male	Medical doctor/ General practitioner	Government hospital, Kampala
8	Female	Researcher and Clinical officer	Government hospital, Kampala
9	Male	Urologist	Cancer Institute, Kampala
10	Female	Surgeon and Cancer researcher	Government hospital, Kampala
	**ROUND 2**		
1	Female	Urologist	Private for-profit hospital, Kampala
2	Female	Surgeon and Cancer researcher	Government hospital, Kampala
3	Female	Researcher and clinical officer	Government hospital, Kampala
4	Male	Medical doctor/ General practitioner	Government hospital, Kampala
5	Male	Urologist	Government hospital, Kampala
6	Male	Medical doctor/ General practitioner	Private not-for-profit hospital, Kampala
7	Female	Radiologist	Private not-for-profit hospital, Kampala
8	Female	Radiologist	Private not-for-profit hospital, Kampala
9	Male	Surgeon	Government hospital, Mukono

For peer review only

10	Male	Medical doctor/ General practitioner	Government hospital, Mukono
11	Male	Surgeon	Private for-profit hospital, Kampala
12	Male	Radiologist	Private not-for-profit hospital, Kampala

Following the first round of the Delphi technique, we excluded three statements of research priorities from the second-round questionnaire where <30% of participants had given them a score less than 15. These statements were related to barriers such as staff motivation, inadequate training etc. Two of the barriers scored greater than 70% and were to be included within our final selection of research priorities. These were "*lack of awareness of cancer as a disease*" and "*poor health care literacy*" (table 2).

Table 2: Ranking of Round 1 questionnaire statements.

	**Statement**	**Ranking (% of participants scoring reason >=15)**
1	Lack of awareness of cancer as a disease and recognition of symptoms	90
2	Poor Healthcare literacy (when, how and where to seek services)	90
3	Lack of critical surgical supplies	70
4	Cost of diagnostic investigations	70
5	Cost of treatments e.g., surgery, radiotherapy, hormone therapy	70
6	Lack of Workforce (basic numbers low and inadequate training of staff)	70
7	Accessibility of care (long distance/travel times to access specialist services)	60
8	Difficulties with healthcare coordination between regions and hospitals as patients referred for specialist investigation and treatment	60
9	Lack of availability of Essential medicines	60
10	Cost of accessing healthcare (e.g., cost of accommodation and transport needed to receive treatment from centralized services)	60

For peer review only - http://bmjopen.bmj.com/site/about/guidelines.xhtml

11	Misdiagnosis of cancer at lower system levels (e.g., primary care, district hospital)	50
12	Lack of Diagnostic services (X-ray, Ultrasound, labs (e.g., PSA testing, biopsy facilities)	50
13	Lack of Radiotherapy options (brachytherapy/ teletherapy)	50
14	Preference for traditional, complementary, and alternative medicine	40
15	Lack of trust in healthcare and patients’ citizens' rights (perceived quality; attitudes of healthcare workers; previous bad experience e.g., patients being turned away or refusal to refer; adequate consent)	40
16	Personal and professional obligations (financial and social implications to the patient and their families of seeking care and undergoing treatment)	40
17	Lack of social capital to support cancer journey especially where patients must travel for care (relationships, support from family, friends, colleagues)	40
18	Patient Fitness and treatment toxicity	40
19	Stigma associated with cancer diagnosis or severe illness/ Fears and beliefs around cancer	30
20	Communication/ language barrier between health care staff and patients	20
21	Staff motivation and burnout	20

For peer review only

In Round 2, of the 16 statements evaluated, four statements reached the consensus target (>70% of respondents scoring the statements >=15) as shown in table 3. These were: (i) Lack of diagnostic services (X-ray, Ultrasound, labs (e.g., PSA testing, biopsy facilities, (ii) Lack of availability of critical medicines, (iii) Lack of Radiotherapy options (brachytherapy/ teletherapy), and (iv) Cost of treatment e.g., surgery, radiotherapy, hormone therapy. In total, six research priorities were identified during the two round Delphi technique.

Table 3: Round 2 ranking

**Rank**	**Barrier**	**Ranking (% of participants**

		scoring reason >=15)
1	Lack of Diagnostic services (X-ray, Ultrasound, labs (e.g., PSA testing, biopsy facilities)	92
2	Lack of availability of critical medicines	92
3	Lack of Radiotherapy options (brachytherapy/ teletherapy)	83
4	Cost of treatment e.g., surgery, radiotherapy, hormone therapy	83
5	Lack of critical Surgical Supplies	67
6	Cost of accessing healthcare (e.g., cost of accommodation and transport needed to receive treatment from centralized services)	67
7	Accessibility of care (long distance/travel times to access specialist services)	58
8	Cost of diagnostic investigations	58
9	Misdiagnosis of cancer at lower system levels (e.g., primary care, district hospital)	50
10	Patient Fitness and treatment toxicity	50
11	Difficulties with healthcare coordination between regions and hospitals as patients referred for specialist investigation and treatment	50
12	Lack of trust in healthcare system and patients' citizens' rights (perceived quality; attitudes of healthcare workers; previous bad experience e.g., patients being turned away or refusal to refer; adequate consent)	33
13	Lack of Workforce (basic numbers low)	33
14	Preference for traditional, complementary, and alternative medicines	25
15	Personal and professional obligations (financial and social implications to the patient and their families of seeking care and undergoing treatment)	25
16	Lack of social capital to support cancer journey especially where patients must travel for care (relationships, support from family, friends, colleagues)	17

For peer review only - http://bmjopen.bmj.com/site/about/guidelines.xhtml

Discussion

The purpose of this Delphi technique was to prioritise key areas for research in prostate cancer in the Ugandan context and will form the basis of recommendation for cancer control planning and research investments. The Delphi process sought to priorities those factors that had the greatest impact on achieving timely access to high quality care, could be investigated empirically and for which interventions could be developed and implemented.

Our study invited cancer experts, clinicians, and policy experts in and outside cancer management and care to evaluate the top research priorities for prostate cancer care in Uganda. The difference in the panels between Round 1 and 2 was favourable as including participants from different healthcare systems, professional backgrounds and specialities created a relatively diverse panel and suitable representation that provided insightful ideas and perspectives towards the priority research needs for prostate cancer in the Ugandan context. There could be potential bias introduced by two different panels participating in the two Delphi rounds as it is often impractical to secure the participation of all key knowledgeable individuals for all the Delphi rounds and consensus meeting. However, we were able to include participants from different health service strata (primary and secondary care), professional backgrounds and specialities to provided insightful perspectives towards prioritising research needs for prostate cancer in the Ugandan context. The priorities chosen reflect important challenges including cancer awareness, increasing diagnostic and treatment capacity as well as addressing the high costs of care, which are not likely to have substantially changed with additional Delphi rounds or participants.

Six key domains were identified: (i) lack of awareness of cancer as a disease and recognition of symptoms, (ii) poor healthcare literacy, (iii) lack of diagnostic services, (iv) lack of availability of essential medicines, (v) lack of radiotherapy facilities (vi) cost of treatment. With respect to these six areas there remains a paucity of literature to date in the Ugandan context but are important issues as outlined below.

Lack of awareness of cancer as a disease and poor recognition of cancer symptoms

Poor knowledge and misconceptions regarding prostate cancer and screening among Ugandan men, (24) is likely to contribute to diagnosis with advanced clinical stages (stage III and IV) of PC. (25) A lack of awareness about cancer and low health literacy has been reported as one of the major reasons for delays in seeking quality cancer care in sub-Saharan

For peer review only - http://bmjopen.bmj.com/site/about/guidelines.xhtml

Africa. (15) Similarly, the lack of awareness of prostate cancer its symptoms and its consequences, definitely affects the uptake of screening and seeking specialist help that provides an opportunity for early detection of prostate cancer. The lack of adequate information and counselling on prostate cancer has been associated with the considerably low uptake of prostate cancer screening among Ugandan men. (26) Developing appropriate interventions for improving awareness and knowledge about prostate cancer are critical in supporting early detection and treatment outcomes of men who develop prostate cancer.

Low health literacy (when, how and where to seek services)

Healthcare literacy is defined as the degree to which individuals have the capacity to obtain, process, and understand basic health information and services needed to make appropriate health decisions and to successfully navigate the health care system. (27, 28) Poor healthcare literacy limits access to care, interaction with health services providers and illness management as people with low healthcare literacy are more likely to have poorer use of health services and therefore experience poorer health outcomes. (29) A recent study among prostate cancer survivors in Uganda has shown that inconsistent information or complete lack of information are common experiences of the prostate cancer survivors can affect their health outcomes. (30, 31) Patients with limited health literacy may have limited knowledge and understanding of health and when coupled with lack of established follow-up mechanisms and inadequate social care support services experienced by many cancer patients in Uganda, this reduces their autonomy in self-care and decision making. (6)

Lack of diagnostic services

A lack of basic imaging and pathological services results in delays in confirming a cancer diagnosis and completing staging to support management options. Such delays have a knock-on effect on the timely delivery of treatment leads to high mortality, morbidity, and low quality of life. (25, 32, 33) Lack of diagnostic services has led to delayed cancer diagnosis being a common in the Uganda setting. (31) Additionally, where the service has been obtained the high costs of diagnostic investigations compounded by the poor social-economic status of the patients has resulted in enormous delays in seeking prostate cancer care among Ugandan men. (6)

Lack of availability of essential medicines

For peer review only - http://bmjopen.bmj.com/site/about/guidelines.xhtml

A recent review has reported major barriers in access to core cancer medicines worldwide, with high prices are major barrier including medicine included in the WHO essential medicines list. (34) Even relatively low cost medicines (compared to chemotherapy and molecularly targeted anti-cancer agents) such as Goserellin are largely unavailable to patients in the Ugandan setting due to recurrent stock-outs and majority being costly. (6) A recent recommendation has suggested increased repurposing of existing drugs such as Metformin, Valproic acid initially intended for other conditions to treat prostate cancer. (35)

Lack of Radiotherapy facilities

This is a core treatment for prostate cancer particularly in high-risk disease. However, radiotherapy is not commonly available in sub Saharan Africa with limited experience for radiotherapy and brachytherapy, insufficient infrastructure as well as limited trained personnel and training opportunities. (36) Its low provision in Uganda has made the provision of treatment unachievable for many cancer sufferers. It has been previously noted that Uganda needs more than 20 operational radiotherapy units in order to respond adequately to its population demands. (37) Currently the only radiotherapy machines in the country are all located at the UCI in the capital city Kampala. This has resulted in demand for services not being met (simply patients do not receive it), prolonged waiting times, compromises timing between the administration of radiation doses and eventually clinical and treatment outcomes. Even when potentially lifesaving radiotherapy treatment options are made available, there are challenges with the cost of treatment for a known cornerstone of curative therapy.

The cost of treatment (surgery, radiotherapy, hormone therapy)

The cost of PC care is critical as there is limited access and availability to safe and reliable services including chemotherapy for prostate cancer patients in Uganda. The government of Uganda provides generic anticancer medicines for patients at the UCI at subsidised costs under the Universal Health Coverage (UHC) strategy to make available the WHO Essential anticancer Medicine List (EML).(38) For example, at the UCI, patients pay a subsidised fee of approximately $85 before accessing radiotherapy services. (6) In general, most cancer patients in Uganda are not able to afford cancer therapy out of pocket and yet the UHC arrangement does not make available newer anticancer therapies. The proportion of anticancer agents especially the newer targeted therapies on the 2019 WHO EML that are available on the 2016 Uganda National Essential Medicines List (NEML) was about 70.5%. Essential medicines for prostate cancer including Leuprolide, Bicalutamide and Abiraterone

For peer review only - http://bmjopen.bmj.com/site/about/guidelines.xhtml

are often out of stock through the government UHC strategy. (39) Availability and prices of anticancer agents vary widely in the LMICs including Uganda. The low availability and unaffordability of anticancer agents often lead patients to turn to alternative care approaches. (40) PC is among the commonest cancers treated with complimentary therapies in Uganda. The unavailability of critical medicines and curative treatments being out of reach has pushed many patients to seek alternative, traditional, Chinese, and complementary medicines chiefly from potential anticancer medicinal plants. (6, 41)

Overall, the study was able to collate and appraise the factors influencing diagnostic and treatment delays in prostate cancer care in Uganda. The study identified specific priority areas that are both high impact (significant cause of avoidable mortality) and modifiable (amenable to health care intervention) to direct resources and interventions to reduce disparities in access to prostate cancer care and improve outcomes. Future research investigating the interaction of the identified barriers and research priorities is necessary to build resilient and effective prostate cancer control programmes for Sub-Saharan Africa. The findings contribute to the multi-country evaluation focusing on prostate cancer to enable strategic priority setting and capacity building tailored for sub-Saharan Africa.

Strengths and limitations

The Delphi technique was conducted by a multidisciplinary team following the CREDES guidelines. (16) The statements explored in the Delphi were identified through a systematic review. We were able to use both electronic web-based as well as face-to-face paper-based approaches to data collection. This study has some limitations: the response rates in round 1 and round 2 were relatively low (34% and 29%, respectively). However, the aim of this study was to have suitable representation to identify the top research priorities for prostate cancer care in Uganda rather than receiving a high response rate.

The discrepancy between the ratings of the statements across the two rounds as indicated above, may have been introduced by two different panels with the differences in professional backgrounds of the participants participating in the two Delphi rounds. The reason for including a broader range of participants was due to the high attrition rate of Panel 1 members This is as a result of the fact that it is often impractical to secure the participation of all key knowledgeable individuals for all the Delphi rounds and consensus meeting. The study team agreed to invite more participants for round 2 by including cancer experts and

For peer review only - http://bmjopen.bmj.com/site/about/guidelines.xhtml

clinicians working in settings beyond those invited in round 1 to include individuals from government hospitals, Private-for-profit hospitals, Private-not-for-profit hospitals, Ministry of Health, and the university sector. Overall, we feel this breadth of participants involving cancer experts, clinicians, policy experts in and outside cancer management to evaluate the top research priorities for prostate cancer care in Uganda is a strength.

Further research is required to elicit patients' views. Although our questionnaires were sent to various groups and healthcare professionals from different clinical roles, most of the participants and experts in this study were oncologists and medical doctors. Future studies may need to consider other members of the healthcare team who were under-represented and may have alternative perspectives.

Conclusion

This study has identified the top six research priorities for prostate cancer in Uganda. Our findings have implications for designing appropriate and contextual prostate care services locally to ensure men have fewer barriers to receiving earlier diagnosis and high-quality affordable treatment and survivorship care.

For peer review only

Acknowledgements:

We thank the participants who participated in the Delphi technique and research team in Uganda where the study was conducted.

Author contributions:

AA conceived the study idea and designed it with ASS, ADM and JS. AA, ASS, ADM, JS, DCL, MM and SM were engaged in the preparation and conduct of the Delphi study. ASS and RM collected the data. ASS, RM, DCL, MM, SM, and AA led the writing of the paper and participated in the analysis. AA, ADM, and JS supervised the overall study. All authors read and approved the final manuscript.

Declaration of conflicting interests:

The author(s) declared no potential conflicts of interest with respect to the research, authorship, and/or publication of this article.

Patient consent for publication:

Not required.

Ethics approval:

Ethical approval was obtained from the Uganda Virus Research Institute Research Ethics Committee (UVRI-REC) Ref: GC/127/21/09/859 and the Uganda National Council for Science and Technology (UNCST) Ref: HS1790ES and the London School of Hygiene and Tropical Medicine Ref: 26672.

Funding:

The study was supported by funding from Wellcome's Institutional Strategic Support Fund grant no. 204928/Z/16/Z through the London School of Hygiene and Tropical Medicine.

Data sharing statement:

All data relevant to the study are included in the article or uploaded as supplementary information.

For peer review only - http://bmjopen.bmj.com/site/about/guidelines.xhtml

References

1. Sung H, Ferlay J, Siegel RL, Laversanne M, Soerjomataram I, Jemal A, et al. Global Cancer Statistics 2020: GLOBOCAN Estimates of Incidence and Mortality Worldwide for 36 Cancers in 185 Countries. CA Cancer J Clin. 2021;71(3):209-49.
2. Fitzmaurice C, Abate D, Abbasi N, Abbastabar H, Abd-Allah F, Abdel-Rahman O, et al. Global, Regional, and National Cancer Incidence, Mortality, Years of Life Lost, Years Lived With Disability, and Disability-Adjusted Life-Years for 29 Cancer Groups, 1990 to 2017: A Systematic Analysis for the Global Burden of Disease Study. JAMA Oncol. 2019;5(12):1749-68.
3. Taitt HE. Global Trends and Prostate Cancer: A Review of Incidence, Detection, and Mortality as Influenced by Race, Ethnicity, and Geographic Location. Am J Mens Health. 2018;12(6):1807-23.
4. Adeloye D, David RA, Aderemi AV, Iseolorunkanmi A, Oyedokun A, Iweala EE, et al. An Estimate of the Incidence of Prostate Cancer in Africa: A Systematic Review and Meta-Analysis. PLoS One. 2016;11(4):e0153496.
5. Cassell A, Yunusa B, Jalloh M, Mbodji MM, Diallo A, Ndoye M, et al. A Review of Localized Prostate Cancer: An African Perspective. World J Oncol. 2019;10(4-5):162-8.
6. Nakaganda A, Solt K, Kwagonza L, Driscoll D, Kampi R, Orem J. Challenges faced by cancer patients in Uganda: Implications for health systems strengthening in resource limited settings. J Cancer Policy. 2021;27:100263.
7. Singh K, Abdel Goad EH, Ramklass SS. Waiting times for prostate cancer diagnosis in KwaZulu-Natal, South Africa. S Afr Med J. 2015;105(6):484-6.
8. Omisanjo OA, Ogunremi OO, Akinola OO, Adebayo OO, Ojewuyi O, Omorinde MO, et al. Waiting Times for Prostate Cancer Diagnosis in a Nigerian Population. J Cancer Epidemiol. 2021;2021:5534683.
9. McGinley KF, Tay KJ, Moul JW. Prostate cancer in men of African origin. Nat Rev Urol. 2016;13(2):99-107.
10. Moodley J, Constant D, Mwaka AD, Scott SE, Walter FM. Mapping awareness of breast and cervical cancer risk factors, symptoms and lay beliefs in Uganda and South Africa. PloS one. 2020;15(10):e0240788.
11. Ilaboya D, Gibson L, Musoke D. Perceived barriers to early detection of breast cancer in Wakiso District, Uganda using a socioecological approach. Global Health. 2018;14(1):9.
12. Ndejjo R, Mukama T, Kiguli J, Musoke D. Knowledge, facilitators and barriers to cervical cancer screening among women in Uganda: a qualitative study. BMJ Open. 2017;7(6):e016282.
13. Wabinga HR, Nambooze S, Amulen PM, Okello C, Mbus L, Parkin DM. Trends in the incidence of cancer in Kampala, Uganda 1991–2010. International journal of cancer. 2014;135(2):432-9.
14. Parkin DM, Nambooze S, Wabwire-Mangen F, Wabinga HR. Changing cancer incidence in Kampala, Uganda, 1991-2006. International journal of cancer. 2010;126(5):1187-95.
15. Lombe DC, Mwamba M, Msadabwe S, Bond V, Simwinga M, Ssemata AS, et al. Delays in seeking, reaching and access to quality cancer care in sub-Saharan Africa: a systematic review. BMJ Open. 2023;13(4):e067715.
16. Jünger S, Payne SA, Brine J, Radbruch L, Brearley SG. Guidance on Conducting and REporting DElphi Studies (CREDES) in palliative care: Recommendations based on a methodological systematic review. Palliat Med. 2017;31(8):684-706.
17. Jones J, Hunter D. Consensus methods for medical and health services research. Bmj. 1995;311(7001):376-80.
18. Boulkedid R, Abdoul H, Loustau M, Sibony O, Alberti C. Using and reporting the Delphi method for selecting healthcare quality indicators: a systematic review. PLoS One. 2011;6(6):e20476.
19. Cotache-Condor C, Kantety V, Grimm A, Williamson J, Landrum KR, Schroeder K, et al. Determinants of delayed childhood cancer care in low- and middle-income countries: A systematic review. Pediatr Blood Cancer. 2023;70(3):e30175.

For peer review only - http://bmjopen.bmj.com/site/about/guidelines.xhtml

20. Pacagnella RC, Cecatti JG, Osis MJ, Souza JP. The role of delays in severe maternal morbidity and mortality: expanding the conceptual framework. Reprod Health Matters. 2012;20(39):155-63.
21. Thaddeus S, Maine D. Too far to walk: maternal mortality in context. Soc Sci Med. 1994;38(8):1091-110.
22. Schneider P, Evaniew N, Rendon JS, McKay P, Randall RL, Turcotte R, et al. Moving forward through consensus: protocol for a modified Delphi approach to determine the top research priorities in the field of orthopaedic oncology. BMJ Open. 2016;6(5):e011780.
23. Harris PA, Taylor R, Minor BL, Elliott V, Fernandez M, O'Neal L, et al. The REDCap consortium: Building an international community of software platform partners. J Biomed Inform. 2019;95:103208.
24. Nakandi H, Kirabo M, Semugabo C, Kittengo A, Kitayimbwa P, Kalungi S, et al. Knowledge, attitudes and practices of Ugandan men regarding prostate cancer. Afr J Urol. 2013;19(4):165-70.
25. Yahaya JJ, Okecha T, Odida M, Wabinga H. Prognostic Factors for Overall Survival of Patients with Prostate Cancer in Kyadondo County, Uganda. Prostate Cancer. 2020;2020:8517130.
26. Ekwan R, Bua E, Nantale R, Opito R, Abingwa P, Sserwanja Q, et al. Uptake of prostate cancer screening and associated factors among men aged 50 years and above in Lira city, Uganda: a cross-sectional study. BMC Public Health. 2023;23(1):432.
27. Hersh L, Salzman B, Snyderman D. Health Literacy in Primary Care Practice. Am Fam Physician. 2015;92(2):118-24.
28. Samerski S. Health literacy as a social practice: Social and empirical dimensions of knowledge on health and healthcare. Soc Sci Med. 2019;226:1-8.
29. Budhathoki SS, Pokharel PK, Good S, Limbu S, Bhattachan M, Osborne RH. The potential of health literacy to address the health related UN sustainable development goal 3 (SDG3) in Nepal: a rapid review. BMC Health Serv Res. 2017;17(1):237.
30. Nabisubi P, Nanyingi M, Okeny PK. Lived experiences of prostate cancer patients below 55 years of age: A phenomenological study of outpatients receiving treatment at the Uganda Cancer Institute. East and Central African Journal of Surgery,. 2020.
31. Bunani N, Kisakye AN, Ssennyonjo A, Nuwaha F. Late Diagnosis among patients with Prostate Cancer at the Uganda Cancer Institute: A retrospective cohort study. Research Square. 2020.
32. Seraphin TP, Joko-Fru WY, Manraj SS, Chokunonga E, Somdyala NIM, Korir A, et al. Prostate cancer survival in sub-Saharan Africa by age, stage at diagnosis, and human development index: a population-based registry study. Cancer Causes Control. 2021;32(9):1001-19.
33. Hanna TP, King WD, Thibodeau S, Jalink M, Paulin GA, Harvey-Jones E, et al. Mortality due to cancer treatment delay: systematic review and meta-analysis. Bmj. 2020;371:m4087.
34. Fundytus A, Sengar M, Lombe D, Hopman W, Jalink M, Gyawali B, et al. Access to cancer medicines deemed essential by oncologists in 82 countries: an international, cross-sectional survey. Lancet Oncol. 2021;22(10):1367-77.
35. Bahmad HF, Demus T, Moubarak MM, Daher D, Alvarez Moreno JC, Polit F, et al. Overcoming Drug Resistance in Advanced Prostate Cancer by Drug Repurposing. Med Sci (Basel). 2022;10(1).
36. Harvey N, Adeyoju A, Brough R. Prostate Cancer in Sub-Saharan Africa: Diagnosis and Management. Cancer in Sub-Saharan Africa: Springer; 2017. p. 95-107.
37. Massoud S. Challenges of making radiotherapy accessible in developing countries. Cancer Control. 2013;2013:85-94.
38. WHO. World Health Organization Model List of Essential Medicines, 21st List, 2019. Geneva: World

Health Organization; 2019. Licence: CC BY-NC-SA 3.0 IGO. Available at: https://appswhoint/iris/bitstream/handle/10665/325771/WHO-MVP-EMP-IAU-201906-engpdf?sequence=1&isAllowed=y Last accessed on 9th May 2023. 2019.
39. Kizub DA, Naik S, Abogan AA, Pain D, Sammut S, Shulman LN, et al. Access to and Affordability of World Health Organization Essential Medicines for Cancer in Sub-Saharan Africa: Examples from Kenya, Rwanda, and Uganda. The oncologist. 2022;27(11):958-70.

For peer review only - http://bmjopen.bmj.com/site/about/guidelines.xhtml

40. Ocran Mattila P, Ahmad R, Hasan SS, Babar Z-U-D. Availability, Affordability, Access, and Pricing of Anti-cancer Medicines in Low- and Middle-Income Countries: A Systematic Review of Literature. Frontiers in Public Health. 2021;9.

41. Omara T, Kiprop AK, Ramkat RC, Cherutoi J, Kagoya S, Moraa Nyangena D, et al. Medicinal Plants Used in Traditional Management of Cancer in Uganda: A Review of Ethnobotanical Surveys, Phytochemistry, and Anticancer Studies. Evid Based Complement Alternat Med. 2020;2020:3529081.

For peer review only

For peer review only - http://bmjopen.bmj.com/site/about/guidelines.xhtml

Guidance Document for Completion of the APC-DOUG Survey

The guidance below relates to Round 1 of the Delphi process.

The aim of Round 1 is to prioritize for further research and intervention development, the major barriers that exist in accessing prostate cancer care. We have considered three aspects to the delays that may occur in receiving a diagnosis and/or treatment.

Delay 1 (seeking care): This is the delay in recognizing illness and deciding to seek appropriate medical help outside the home.

Delay 2 (reaching care): This is the delay in reaching an appropriate health facility.

Delay 3 (receiving quality care): This is the delay in receiving quality care after reaching the health facility.

We are kindly asking you to review and answer the questions below based on your experience.

Instructions:

1. There are 21 statements identified from a review of the wider empirical literature as a reason for delay in accessing cancer care.
2. We want to understand how important or relevant these reasons for delays are with respect to prostate cancer in Uganda
3. Each statement will be scored out of 20 based on 4 assessment domains a. feasibility to study this issue; b. scale of the issue; c. impact of the barrier on prostate cancer outcomes; d. feasibility of addressing this barrier
4. Please score each domain on each delay out of 5, with 5 being the highest (most important) and 1 being the least (important).
5. The statements assigned the highest scores will be considered as main priorities for round 2 of the Delphi process.

If you have any questions, please contact Dr Amos D. Mwaka email: mwakaad@yahoo.com or Andrew S. Ssemata email: andrew.ssemata@mrcuganda.org.

For peer review only - http://bmjopen.bmj.com/site/about/guidelines.xhtml

Reason for delay	Feasible (Easy to measure or investigate this reason for delay)	Large scale (This delay affects a significant proportion of prostate cancer patients)	High Impact (This delay is a significant or avoidable cause of death or disability from prostate cancer)	Modifiable (The reason identified can be readily changed to improve care)
	Please score each theme for each statement with a score of 1 – 5 based on the explanation in brackets (1 is lowest and 5 is highest)			
1. Accessibility of care (long distance/travel times to access specialist services)				
2. Lack of awareness of cancer as a disease and recognition of symptoms				
3. Poor healthcare literacy (when, how and where to seek services)				
4. Preference for traditional, complementary, and alternative medicines				
5. Lack of trust in healthcare system and patients' citizens' rights (perceived quality; attitudes of healthcare workers; previous bad experience e.g., patients being turned away or refusal to refer; adequate consent)				
6. Personal and professional obligations (financial and social implications to the patient and their families of seeking care and undergoing treatment)				
7. Stigma associated with a cancer diagnosis or severe illness/ Fears and beliefs around cancer				
8. Lack of social capital (relationships, support from family, friends, colleagues)				

For peer review only - http://bmjopen.bmj.com/site/about/guidelines.xhtml

For peer review only

Reason for delay	Feasible (Easy to measure or investigate this reason for delay)	Large scale (This delay affects a significant proportion of prostate cancer patients)	High Impact (This delay is a significant or avoidable cause of death or disability from prostate cancer)	Modifiable (The reason identified can be readily changed to improve care)
9. Misdiagnosis of cancer (at lower system levels e.g. primary care, district hospital)				
10. Difficulties with healthcare coordination between regions and hospitals as patients referred for specialist investigation and treatment				
11. Lack of diagnostic services (X-ray, Ultrasound, labs (e.g. PSA testing, biopsy facilities)				
12. Lack of availability of critical medicines				
13. Lack of radiotherapy options (brachytherapy/ teletherapy)				
14. Lack of critical surgical supplies				
15. Cost of accessing healthcare (e.g., cost of accommodation and transport needed to receive treatment from centralized services)				
16. Cost of diagnostic investigations				
17. Cost of treatments e.g. surgery, radiotherapy. Hormone therapy				
18. Lack of workforce (basic numbers low and inadequately trained staff)				
19. Patient fitness and treatment toxicity				
20. Communication/language barriers between health care staff and patients				
21. Staff motivation and burnout				

For peer review only

For peer review only - http://bmjopen.bmj.com/site/about/guidelines.xhtml

For peer review only

For peer review only - http://bmjopen.bmj.com/site/about/guidelines.xhtml

Guidance Document for Completion of the APC-DOUG Survey

The guidance below relates to Round 2 of the Delphi process.

The aim of Round 2 is to prioritize for further research and intervention development, the major barriers that exist in accessing prostate cancer care. We have considered three aspects to the delays that may occur in receiving a diagnosis and/or treatment.

Delay 1 (seeking care): This is the delay in recognizing illness and deciding to seek appropriate medical help outside the home.

Delay 2 (reaching care): This is the delay in reaching an appropriate health facility.

Delay 3 (receiving quality care): This is the delay in receiving quality care after reaching the health facility.

We are kindly asking you to review and answer the questions below based on your experience.

Instructions:

1. There are 18 statements identified from a review of the wider empirical literature as a reason for delay in accessing cancer care.
2. We want to understand how important or relevant these reasons for delays are with respect to prostate cancer in Uganda
3. Each statement will be scored out of 20 based on 4 assessment domains a. feasibility to study this issue; b. scale of the issue; c. impact of the barrier on prostate cancer outcomes; d. feasibility of addressing this barrier
4. Please score each domain on each delay out of 5, with 5 being the highest (most important) and 1 being the least (important).
5. The statements assigned the highest scores will be considered as main priorities for round 2 of the Delphi process.

If you have any questions, please contact Dr Amos D. Mwaka email: mwakaad@yahoo.com or Andrew S. Ssemata email: andrew.ssemata@mrcuganda.org.

For peer review only - http://bmjopen.bmj.com/site/about/guidelines.xhtml

Reason for delay	Feasible (Easy to measure or investigate this reason for delay)	Large scale (This delay affects a significant proportion of prostate cancer patients)	High Impact (This delay is a significant or avoidable cause of death or disability from prostate cancer)	Modifiable (The reason identified can be readily changed to improve care)
	Please score each theme for each statement with a score of 1 – 5 based on the explanation in brackets (1 is lowest and 5 is highest)			
1. Accessibility of care (long distance/travel times to access specialist services)				
2. Lack of awareness of cancer as a disease and recognition of symptoms				
3. Poor healthcare literacy (when, how and where to seek services)				
4. Preference for traditional, complementary, and alternative medicines				
5. Lack of trust in healthcare system and patients' citizens' rights (perceived quality; attitudes of healthcare workers; previous bad experience e.g., patients being turned away or refusal to refer; adequate consent)				
6. Personal and professional obligations (financial and social implications to the patient and their families of seeking care and undergoing treatment)				
7. Lack of social capital (relationships, support from family, friends, colleagues)				
Reason for delay	Feasible (Easy to measure or investigate this reason for delay)	Large scale (This delay affects a significant proportion of prostate cancer patients)	High Impact (This delay is a significant or avoidable cause of death or disability from prostate cancer)	Modifiable (The reason identified can be readily changed to improve care)

For peer review only

For peer review only - http://bmjopen.bmj.com/site/about/guidelines.xhtml

8. Misdiagnosis of cancer (at lower system levels e.g. primary care, district hospital)				
9. Difficulties with healthcare coordination between regions and hospitals as patients referred for specialist investigation and treatment				
10. Lack of diagnostic services (X-ray, Ultrasound, labs (e.g. PSA testing, biopsy facilities)				
11. Lack of availability of critical medicines				
12. Lack of radiotherapy options (brachytherapy/ teletherapy)				
13. Lack of critical surgical supplies				
14. Cost of accessing healthcare (e.g., cost of accommodation and transport needed to receive treatment from centralized services)				
15. Cost of diagnostic investigations				
16. Cost of treatments e.g. surgery, radiotherapy. Hormone therapy				
17. Lack of workforce (basic numbers low and inadequately trained staff)				
18. Patient fitness and treatment toxicity				

For peer review only

For peer review only - http://bmjopen.bmj.com/site/about/guidelines.xhtml

Reporting checklist for cross sectional study.

Based on the STROBE cross sectional guidelines.

Instructions to authors

Complete this checklist by entering the page numbers from your manuscript where readers will find each of the items listed below.

Your article may not currently address all the items on the checklist. Please modify your text to include the missing information. If you are certain that an item does not apply, please write "n/a" and provide a short explanation.

Upload your completed checklist as an extra file when you submit to a journal.

In your methods section, say that you used the STROBE cross sectionalreporting guidelines, and cite them as:

von Elm E, Altman DG, Egger M, Pocock SJ, Gotzsche PC, Vandenbroucke JP. The Strengthening the Reporting of Observational Studies in Epidemiology (STROBE) Statement: guidelines for reporting observational studies.

For peer review only

		Reporting Item	Page Number
**Title and abstract**			
Title	#1a	Indicate the study's design with a commonly used term in the title or the abstract	1
Abstract	#1b	Provide in the abstract an informative and balanced summary of what was done and what was found	2
**Introduction**			
Background / rationale	#2	Explain the scientific background and rationale for the investigation being reported	3-4
Objectives	#3	State specific objectives, including any prespecified hypotheses	4
**Methods**			
Study design	#4	Present key elements of study design early in the paper	4
Setting	#5	Describe the setting, locations, and relevant dates, including periods	5

		of recruitment, exposure, follow-up, and data collection	
Eligibility criteria	#6a	Give the eligibility criteria, and the sources and methods of selection of participants.	6
	#7	Clearly define all outcomes, exposures, predictors, potential confounders, and effect modifiers. Give diagnostic criteria, if applicable	6
Data sources / measurement	#8	For each variable of interest give sources of data and details of methods of assessment (measurement). Describe comparability of assessment methods if there is more than one group. Give information separately for for exposed and unexposed groups if applicable.	6
Bias	#9	Describe any efforts to address potential sources of bias	6
Study size	#10	Explain how the study size was arrived at	N/A purposive sample
Quantitative variables	#11	Explain how quantitative variables were handled in the analyses. If applicable, describe which groupings were chosen, and why	6
Statistical methods	#12a	Describe all statistical methods, including those used to control for confounding	6
Statistical methods	#12b	Describe any methods used to examine subgroups and interactions	N/A
Statistical methods	#12c	Explain how missing data were addressed	N/A
Statistical methods	#12d	If applicable, describe analytical methods taking account of sampling strategy	N/A
Statistical methods	#12e	Describe any sensitivity analyses	N/A
**Results**			
Participants	#13a	Report numbers of individuals at each stage of study—eg numbers potentially eligible, examined for eligibility, confirmed eligible, included in the study, completing follow-up, and analysed. Give information separately for for exposed and unexposed groups if	6

For peer review only

		applicable.	
Participants	#13b	Give reasons for non-participation at each stage	7
Participants	#13c	Consider use of a flow diagram	N/A
Descriptive data	#14a	Give characteristics of study participants (eg demographic, clinical, social) and information on exposures and potential confounders. Give information separately for exposed and unexposed groups if applicable.	7-8
Descriptive data	#14b	Indicate number of participants with missing data for each variable of interest	N/A
Outcome data	#15	Report numbers of outcome events or summary measures. Give information separately for exposed and unexposed groups if applicable.	N/A
Main results	#16a	Give unadjusted estimates and, if applicable, confounder-adjusted estimates and their precision (eg, 95% confidence interval). Make clear which confounders were adjusted for and why they were included	N/A
Main results	#16b	Report category boundaries when continuous variables were categorized	7-11
Main results	#16c	If relevant, consider translating estimates of relative risk into absolute risk for a meaningful time period	7-11
Other analyses	#17	Report other analyses done—e.g., analyses of subgroups and interactions, and sensitivity analyses	N/A
**Discussion**			
Key results	#18	Summarise key results with reference to study objectives	11
Limitations	#19	Discuss limitations of the study, taking into account sources of potential bias or imprecision. Discuss both direction and magnitude of any potential bias.	14
Interpretation	#20	Give a cautious overall interpretation considering objectives, limitations, multiplicity of analyses, results from similar studies, and other relevant evidence.	14
Generalisability	#21	Discuss the generalisability (external validity) of the study results	14

For peer review only

Other Information

Funding	#22	Give the source of funding and the role of the funders for the present study and, if applicable, for the original study on which the present article is based	15

Notes:

- 10: N/A purposive sample The STROBE checklist is distributed under the terms of the Creative Commons Attribution License CC-BY. This checklist was completed on 17. May 2023 using https://www.goodreports.org/, a tool made by the EQUATOR Network in collaboration with Penelope.ai

For peer review only
